# REmoval of cytokines during CArdiac surgery (RECCAS): a randomised controlled trial

**DOI:** 10.1186/s13054-024-05175-9

**Published:** 2024-12-12

**Authors:** Andreas Hohn, Nathalie M. Malewicz-Oeck, Dirk Buchwald, Thorsten Annecke, Peter K. Zahn, Andreas Baumann

**Affiliations:** 1 Faculty of Medicine, University of Cologne, Kerpener Str. 62, 50937 Cologne, Germany; 2 Department of Anesthesiology and Intensive Care Medicine, Cologne University Hospital, Kerpener Str. 62, 50937 Cologne, Germany; 3 Department of Anaesthesiology and Intensive Care Medicine, Kliniken Maria Hilf GmbH, Viersener Str. 450, 41063 Moenchengladbach, Germany; 4 Department of Anaesthesiology, Intensive Care Medicine and Pain Medicine, Medical Faculty of Ruhr-University Bochum, BG University Hospital Bergmannsheil gGmbH, Bürkle-de-la-Camp-Platz 1, 44789 Bochum, Germany; 5 Department of Cardiothoracic Surgery, BG University Hospital Bergmannsheil, Bürkle-de-la-Camp-Platz 1, 44789 Bochum, Germany; 6 Department of Anaesthesiology and Intensive Care Medicine, Kliniken der Stadt Köln GmbH, University of Witten Herdecke, Cologne, Ostmerheimer Straße 200, 51109 Cologne, Germany

**Keywords:** Haemoadsorption, Cardiac Surgery, Inflammation, Heart–lung-machine, Cardiopulmonary bypass, Cytokines

## Abstract

**Background:**

Cardiopulmonary bypass (CPB) triggers marked cytokine release often followed by a systemic inflammatory response syndrome, associated with adverse postoperative outcomes. This trial investigates the intraoperative use of haemoadsorption (HA) during cardiac surgery with CPB to assess its impact on postoperative systemic inflammatory response.

**Methods:**

In this prospective randomised controlled trial (ethics approval no. 5094-14DRKS00007928), patients (> 65 years) undergoing elective on-pump cardiac surgery were randomised to intraoperative HA (CytoSorb) during CPB or standard care without HA. Primary outcome was the difference in mean interleukin (IL)-6 serum concentrations between groups on intensive care unit (ICU) admission. The secondary outcomes included various clinical and biochemical endpoints. Statistical methods included paired and unpaired t-tests, Wilcoxon, Mann–Whitney U-tests, and chi-square tests.

**Results:**

Thirty-eight patients were allocated to receive either intraoperative HA (n = 19) or standard care (n = 19). The primary outcome, IL-6 levels on ICU admission, did not differ between the study group and controls (214.4 ± 328.8 vs. 155.8 ± 159.6 pg/ml, *p* = 0.511). During surgery pre- versus post-adsorber IL-2, IL-6, IL-8, IL-10, heparan sulfate and myoglobin post- levels were reduced. Furthermore, IL-6 levels did not differ between the study groups on day 1 and 2 in the ICU. While sequential organ failure assessment scores, lactate levels, and C-reactive protein and procalcitonin (PCT) showed no statistically significant differences. Regarding haemodynamic stability in the treatment group the cardiac index (3.2 ± 0.7 vs. 2.47 ± 0.47 l/min/m^2^, *p* = 0.012) on ICU day 2 increased, and lower fluid requirements as well as decreased fibrinogen requirement were observed. Need for renal replacement therapy did not differ though a shorter duration was observed in the treatment group. Time on ventilator, respiratory parameters, infectious complications, delirium scores, ICU and hospital lengths of stay, and mortality did not differ between groups.

**Conclusion:**

HA did not reduce the IL-6 level on ICU admission or afterwards. Even though HA reduced cytokine load during cardiac surgery in the treatment group. There were no significant differences between groups in the postoperative course of other cytokine concentrations, organ dysfunction, ICU and hospital lengths of stay and mortality rates.

*Trial registration* prospectively DRKS00007928 and published under: Baumann A, Buchwald D, Annecke T, Hellmich M, Zahn PK, Hohn A. RECCAS - REmoval of Cytokines during Cardiac Surgery: study protocol for a randomised controlled trial. Trials. 2016;17: 137.

**Graphical abstract:**

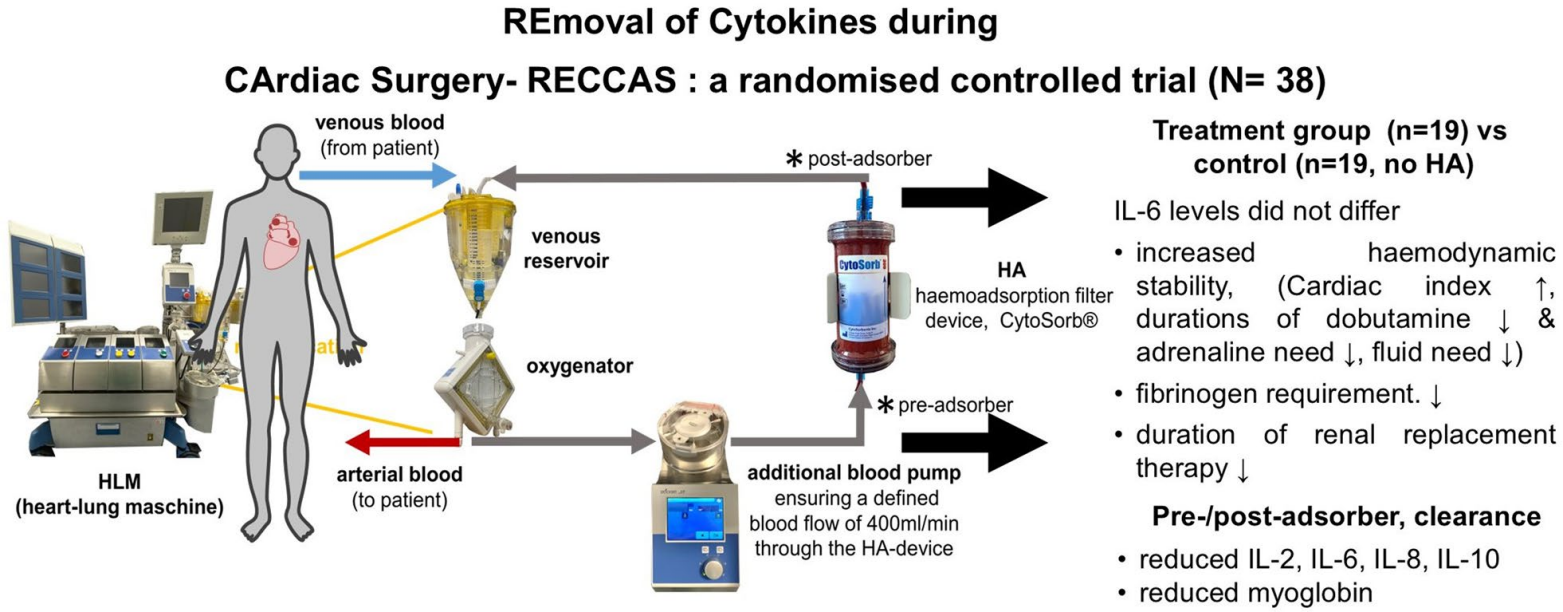

**Supplementary Information:**

The online version contains supplementary material available at 10.1186/s13054-024-05175-9.

## Introduction

On-pump cardiac surgery initiates a substantial release of cytokines inducing a systemic inflammatory response syndrome (SIRS). Concentration of the proinflammatory cytokines interleukin (IL)-6, IL-8, and tumour necrosis factor (TNF)-alpha peak after the cessation of cardiopulmonary bypass (CPB) and gradually recover within 24 h [1]. Systemic inflammation following cardiac surgery is associated with multiorgan dysfunction and significant complications [1–4]. The release of these proinflammatory cytokines correlates with poor postoperative outcomes [5]. In particular, IL-6 has been linked to postoperative myocardial ischaemia, low cardiac output, and the need for vasopressor support [6, 7]. Similarly, elevated IL-6 levels on admission to the intensive care unit (ICU) following cardiac surgery are strongly associated with the emergence of postoperative infections [8]. Despite attempts at interventions such as leukocyte filtration, endotoxin adsorption, and ultrafiltration during CPB, consistent removal of cytokines or inflammatory mediators has not been conclusively demonstrated. While the intraoperative use of adsorption and filtration devices have proven safe and well-tolerated, clinical studies have failed to reveal positive effects on outcomes [9–13].

The extracorporeal sorbent haemoadsorption (HA) device, CytoSorb®, has gained approval in the European Union (EU) for removing elevated cytokine levels in various clinical situations. A systematic review and meta-analysis suggested that among the current adsorbing techniques CytoSorb® appears to be the most promising based on data from animal studies [14–16] and initial clinical results [17]. HA was found to be safe and well-tolerated in more than 2800 human treatments in over 1400 critically ill patients [18].

Limited data currently exist on the ability of CytoSorb® to mitigate the inflammatory response following cardiac surgery [12, 19, 20]. This prospective blinded randomised controlled trial investigated whether the intraoperative use of CytoSorb® in the CPB circuit could effectively reduce postoperative proinflammatory cytokine levels, especially IL-6, and attenuate the systemic inflammatory response leading to favourable outcomes.

## Methods

### Study design

We conducted a prospective randomised, blinded controlled intervention trial. The detailed study protocol has been published previously [21]. Patients were randomised to either the intervention group, receiving HA during CPB inserted in the heart lung machine or to a control group receiving standard CPB without HA [21].

### Ethics

Ethical approval for this prospective single-centre randomised controlled interventional trial was provided by the Ethical Committee of Ruhr University Bochum, Bochum, Germany, on 17.10.2014 (Ethical Committee No. 5094-14) and was registered shortly after beginning of recruitment and prior to completion of recruitment (DRKS00007928), the protocol published [21] and followed the CONSORT statement for RCT’s (supplemental Table 1) [22]. The study was conducted between 2015 and 2019 at University Hospital Bergmannsheil Bochum, Germany, in accordance with the Helsinki Declaration and the International Council for Harmonisation—Guideline for Good Practice ICH-GCP guidelines.

### Enrollment and study procedure

**Fig. 1:**
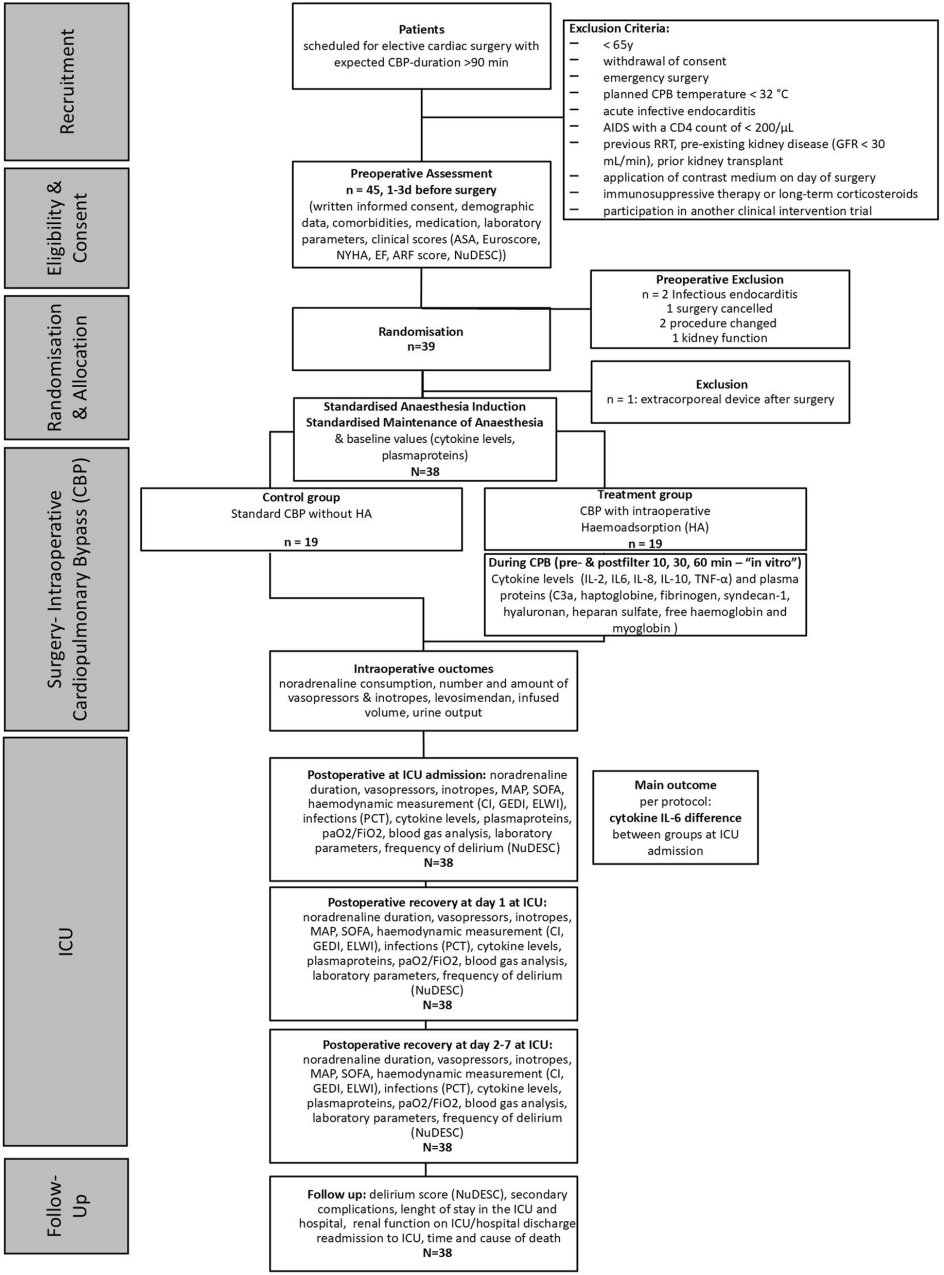
Flow Diagram of Study Design. Consolidated Standards of Reporting Trials (CONSORT) flow diagram of the study design depicting recruiting and dropouts of the REmoval of Cytokines during CArdiac Surgery [2] trial. N = 38. *Abbreviations* C3a, complement component 3a; CPB, cardiopulmonary bypass; HA, haemoadsorption, AIDS, acquired immunodeficiency syndrome; ARF score, acute renal failure score; ASA, American Society of Anesthesiologists; CBP, cardiopulmonary bypass; CD4, cluster of differentiation 4, Cl, cardiac output; EF, ejection fraction, EuroSCORE, European System for Cardiac Operative Risk Evaluation; GEDI, Global End-Diastolic Volume Index, GFR, glomerular filtration rate; HA, haemoadsorption; ICU, intensive care unit; IL, interleukin, MAP, mean arterial pressure; NuDESC, Nursing Delirium Screening Scale; NYHA, New York Heart Association; PCT, procalcitonin; RRT, renal replacement therapy, SOFA, sequential organ failure assessment; TNF-a, tumor necrosis factor alpha; PaO_2_/FiO_2_, ratio of arterial oxygen partial pressure to fractional inspired oxygen

All adult patients (> 65 years) scheduled for elective on-pump cardiac surgery with an expected CPB duration > 90 min at University Hospital Bergmannsheil Bochum in Germany between January 2015 and February 2019 were consecutively screened and recruited during the preoperative visit the day before surgery. Due to standardisation, the selection of study days depended on the availability of a perfusionist trained on the use of HA on the HLM approved for the inclusion of the HA in the CPB circuit. After obtaining written informed consent, patients were randomised, assessed and baseline parameters obtained prior to surgery, during surgery and daily on the ICU for up to 7 days. The details of the gathered information, parameters, and exclusion criteria are displayed in Fig. 1.

### Randomisation and blinding

Randomised block allocation 1:1 using opaque envelopes was performed after induction of anaesthesia before the beginning of surgery, and patients allocated to the intervention (HA application) or control group. The study personnel were trained to collect the data in a standardised manner. Patients, surgeons, ICU team, and lab analysts were blinded to the allocation.

### Standardisation of anaesthesia, CPB management and ICU treatment

Surgery was performed under general anaesthesia. Both groups of patients received standardised anaesthetic treatment. Induction and general anaesthesia was provided by a senior anaesthetist based on the institution’s standard perioperative protocol [23, 24]. Anaesthesia was maintained with inhaled isoflurane or sevoflurane and continuous sufentanil. Patients received cefuroxime (200 mg/kg) prior to surgery and before the initiation of cardiopulmonary bypass.

Tranexamic acid bolus (30 mg/kg) followed by a continuous infusion of 2 mg/kg/h was given for antifibrinolytic prophylaxis until the end of the procedure. For CPB, a bolus of heparin (400 IU/kg) was administered to reach an activated clotting time (ACT) of > 400 s. After CPB and heparin reversal with protamine (300 IU/kg), coagulation therapy was based on thromboelastometry measurements.

Intraoperative transoesophageal echocardiography was performed for optimisation of haemodynamic management and guiding the application of vasopressors and/or inotropic support. Noradrenaline was administered as the first-line vasopressor.

The extracorporeal circulation was performed using a S5 heart–lung-machine (LivaNova PLC, London, United Kingdom). The system included a capillary oxygenator type Quadrox-i (Getinge Deutschland GmbH, Rastatt, Germany) and a Remowell 2 cardiotomy reservoir (Eurosets, Medolla, Italy). A blood flow index of 2.4 l/min/m^2^ was used consistently for all patients. The open perfusion system included a suction device, a vent and a 4:1 blood cardioplegia system according to Buckberg. All perfusions occurred under normothermic conditions. In the treatment group, a specialized heart–lung machine setup was implemented to optimize the flow rate through the cytokine adsorber. This was achieved by incorporating a separate blood pump, which maintained a constant blood flow of 400 ml/min from the start of the bypass. The pump was positioned between the oxygenator and the venous reservoir, ensuring that natural fluctuations in the heart–lung machine’s flow did not affect the blood flow through the adsorber.

Standard of care treatment in the ICU was provided. In particular, mechanical ventilation, nutrition, sedation, anticoagulation, and blood glucose control therapy were based on local treatment protocols. Vasopressors, inotropes and fluid management in the ICU were guided by haemodynamic monitoring via a transpulmonary thermodilution technique (PiCCO®) and the derived dynamic parameters, and or echocardiography (transthoracic or transoesophageal). Renal replacement therapy (RRT), performed as continuous veno-venous haemodialysis (CVVHD) (30 mL/kg/h) with regional citrate-anticoagulation, was based on absolute indications according to Kidney Disease Improving Global Outcomes (KDIGO), including life-threatening refractory changes in fluid, electrolyte and acid–base balance or acute kidney injury (AKI) with urine output < 0.3 mL/kg/ h for ≥ 24 h or anuria for ≥ 12 h (Acute Kidney Injury Network (AKIN) 3) or AKIN 2 with concomitant organ failure (development or progression of non-renal sequential organ failure assessment (SOFA) organ system subscore ≥ 2) and/or haemodynamic instability (noradrenaline /adrenaline ≥ 0.1 μg/kg/min or need of terlipressin). RRT was discontinued if renal recovery occurred (urine output > 400 mL/24 h and creatinine clearance > 20 mL/min) [25].

In the ICU, the patient remained sedated until considered stable, then weaning was initiated, propofol infusion was discontinued and the patient was allowed to breathe in an assisted mode, gradually reducing the oxygen concentration as well as provided level of assistance. Extubation was aimed for as soon as possible.

### The haemoadsorption device (HA, CytoSorb®)

The CytoSorb® adsorber represents a CE-marked Class IIb medical device in accordance with ISO 13485 standards. It is designed for intraoperative use during cardio-pulmonary bypass surgery and is further indicated for the removal of cytokines, bilirubin, and myoglobin from the circulatory system. Comprised of a biocompatible and haemocompatible porous polymer sorbent bead technology, this device demonstrates a notable binding capacity, effectively reducing a diverse array of cytokines and inflammatory mediators. The biocompatibility of the HA-device has been systematically assessed according to ISO10993 guidelines.

Functionally, the adsorber operates through pore capture and surface adsorption mechanisms within whole blood. Characterised by a hydrophobic surface area covering approximately 45,000 m^2^, the device can remove molecules ranging from 5 to 60 kDa, with the efficacy of removal being concentration dependent. This HA device has been specifically tailored to address substances falling within this molecular weight range, aligning with the predominant size spectrum of cytokines and inflammatory mediators. It is important to note that the technology does not rely on affinity-based sorbent methods and does not employ antibodies, ligands, cells, or pharmaceutical agents [14, 26–28].

### Outcome measures

Clinical data and data on outcome parameters were documented in a paper case report form (CRF) initially and finally transferred to REDCap (Research Electronic Data Capture). Both will be stored for at least 10 years according to the ICH–GCP guidelines. The primary outcome was the difference in mean IL-6 serum levels between the two study groups upon admission to the ICU.

According to the study protocol, blood samples were drawn at baseline after the insertion of the arterial line and before induction. In the HA treatment group, additional blood for analysis of cytokine and glycocalyx components such as heparan sulphate, syndecan-1 and hyaluronan was drawn from the CPB circuit before entering (pre-) and after passage through the HA device (post-adsorber) at 10, 30 and 60 min after CPB initialization for analysis of cytokine concentrations and other parameters (supplement Table 2). The cytokine concentration in the blood samples were analyzed to monitor the increase in cytokines during surgery and to measure the clearance of the adsorber.

The next blood sample was drawn on arrival to the ICU as well as on days one (d1) and two (d2) after surgery, at the same time in the morning. During the entire stay secondary parameters were documented and continued until the patient was discharged from ICU. Secondary outcome parameters are provided in supplement Table 2.

All blood samples were immediately centrifuged for 10 min at 2000/min immediately, pipetted, and frozen at − 80 °C until further analysis. For further details, see protocol [21].

### Statistical analyses

The study data were collected and managed using REDCap electronic data capture tools. REDCap [29, 30] data were analysed as prespecified in the protocol [21].

Microsoft Excel (Office2016, MicrosoftCorp., Redmond, USA) and STATA15 (Version 15.1) were used for statistical and GraphPadPrism8 (GraphPad Software) for graphical analysis. Data analysis and dissemination were delayed because of logistical, personnel, and administrative challenges during the COVID-19 pandemic. Unless otherwise stated, all data are presented as means ± standard deviation (SD) for quantitative variables or median (interquartile range (IQR)). Considering *p* < 0.05 as statistically significant and after surveying normality, an unpaired t-test or Mann–Whitney-U-test was performed to compare quantitative variables, and a chi-square-test or Fisher’s exact test (group numbers < 5) for qualitative variables were applied. In cases of pre- and post-data from the same group a Wilcoxon test if normality was not passed (Kolmogorov–Smirnov) or a paired t-test were applied. During HA he clearance of cytokines was calculated as clearance = blood flow* [(cytokine_pre_—cytokine_post_)/cytokine_pre_] based on pre- versus post-adsorber blood cytokine concentrations for a blood flow of 400 ml/min at 10, 30 and 60 min after HA initiation.

In the prespecified sample size calculation [21] based on previous research we expected a mean IL-6 concentration of 200 ± 50 pg/mL in the control group [8, 31–33] and a reduction of 30% to 140 ± 35 pg/mL by the use of intraoperative HA, leading to a required n = 15 per group, n = 38 analysed by two-sided t-test and reaching a power of 95% at α-level of 5% (Stata 15.1; StataCorp, College Station, TX, USA). To compensate in case of potential dropouts 20 patients per group were aimed for. Since at n = 19 only one dropout occurred after randomisation (Fig. 1) the needed sample size was evaluated as reached and sufficient.

## Results

### Patients’ characteristics

Initially 45 patients were enrolled and 38 (age: 74.6 ± 5.5 years, male: 24 (63.2%); Table 1) stayed in the study (dropouts and recruitment in Fig. 1). All surgeries were elective, no emergency surgeries were included.

**Table 1:** Patients´ characteristics N = 38

	Total	Control	Treatment	*p*-Value; MD [CI]
Number (n)	38	19	19	
Male, n (%)	24 (63.2)	13 (68.4)	11 (57.9)	0.501
Age, years, mean ± SD (IQR)	74.6 ± 5.5 (70.3–79.8)	75.1 ± 5.4 (71.5–79.5)	74.1 ± 5.9 (70–78)	0.587; 1 [− 2.7;4.7]
Height, cm, mean ± SD (IQR)	168.7 ± 10 (161.8–176.8)	170.1 ± 9.1 (164–178)	167.4 ± 11.2 (158–176)	0.422; 2.68 [− 4.02;9.39]
Weight, kg, mean ± SD (IQR)	84.5 ± 19.3 (74–94.3)	84 ± 18.8 (75–91)	85.1 ± 20.7 (68–97.5)	0.871; − 1.05 [− 14.08;11.98]
BMI, mean ± SD(IQR)	29.5 ± 5.5 (21.3–50.8)	29 ± 6.2 (21.3–50.8)	29.9 ± 4.9 (23–40.4)	0.595; − 0.97 [− 4.64;2.7]
Euro-Score, %, mean ± SD (IQR)	9 ± 2.8 (7–10.8)	8.9 ± 2.9 (8–10)	9.1 ± 2.9 (6.5–11.5)	0.867; − 0.16 [− 2.06;1.74]
ASA, n (%)				0.547
3	3 (7.9)	1 (5.3)	2 (10.5)	
4	35 (92.1)	18 (94.7)	17 (89.5)	
NYHA, n (%)				0.335
NYHA 1	2 (5.3)	1 (5.3)	1 (5.3)	
NYHA 2	13 (34.2)	4 (21.1)	9 (47.4)	
NYHA 3	17 (44.7)	11 (57.9)	6 (31.6)	
NYHA 4	6 (15.8)	3 (15.8)	3 (15.8)	
EF (ejection fraction) < 35%, n (%)	6 (15.8)	3 (15.8)	3 (15.8)	1.000
Type of surgery, n (%)				0.723
Bypass	5 (13.2)	2 (10.5)	3 (15.8)	
Heart valves replacement/reconstruction	6 (15.8)	2 (10.5)	4 (21.1)	
Combination	10 (26.3)	6 (31.6)	4 (21.1)	
Other	17 (44.7)	9 (47.4)	8 (42.1)	
Duration of surgery, min, mean ± SD(IQR)	323.8 ± 108.2 (170–722)	331.2 ± 93 (193–722)	316.4 ± 121 (170–522)	0.682 14.84 [− 58.13;87.82]
Duration of cardiopulmonary bypass, min, mean ± SD (IQR)	162.6 ± 87.6 (70–559)	163.4 ± 61.7 (85–559)	161.8 ± 107.4 (70–329)	0.957 1.58 [− 57.62;60.78]
Comorbidities, n (%)				
Diabetes mellitus (insulin-dependent)	7 (18.4)	2 (10.5)	5 (26.3)	0.219
Stroke	2 (5.3)	(0)	2 (10.5)	0.292
Liver dysfunction	4 (10.5)	2 (10.5)	2 (10.5)	0.703
Renal insufficiency	37 (97.4)	19 (100)	18 (94.7)	0.468
Stage 1 (GFR > 89 ml/min)	1 (2.6)	(0)	1 (5.3)	0.772
Stage 2 (GFR 60–89 ml/min)	13 (34.2)	7 (36.8)	6 (31.6)	
Stage 3 (GFR 30–59 ml/min)	23 (60.5)	12 (63.2)	11 (57.9)	
Coagulopathy	9 (23.7)	5 (26.3)	4 (21.1)	0.703
Cardiovascular diseases				
Congestive heart failure	15 (39.5)	6 (31.6)	9 (47.4)	0.319
Cardiac arrhythmias	19 (50)	11 (57.9)	8 (42.1)	0.33
Previous cardiac surgery	6 (15.8)	3 (15.8)	3 (15.8)	1.000
SOFA Score at ICU admission, mean ± SD (IQR)	7.8 ± 1.9 (7–9)	7.5 ± 1.9 (7–8.5)	8.2 ± 2 (7–10)	0.287; − 0.68 [0.6;− 1.97]
ARF Score, mean ± SD (IQR)	4.1 ± 1.6 (3–5.8)	3.7 ± 1.7 (2.5–5)	4.4 ± 1.6 (4–6)	0.246; − 0.63 [0.45;− 1.72]
NuDesc Score, mean ± SD (IQR)	0 ± 0 (0–0)	0 ± 0 (0–0)	0 ± 0 (0–0)	
Cytokine levels and plasmaproteins				
IL-6, pg/ml, mean ± SD (IQR)	13.8 ± 8.2 (9.2–15.7)	13.7 ± 6.9 (9.2–16)	13.8 ± 9.8 (8.9–15.7)	0.715^#^ − 0.15 [5.42;− 5.72]
IL-2, pg/ml, mean ± SD (IQR)	10 ± 3 (7.6–13)	8.6 ± 2 (7.6–8.3)	11.4 ± 3.2 (7.9–14.2)	0.026^#^; − 2.87 [− 1.11;− 4.62]
IL-8, pg/ml, mean ± SD (IQR)	13 ± 4.9 (9.8–15.2)	13.5 ± 4.4 (10.6–15.5)	12.6 ± 5.5 (9.6–14.7)	0.457^#^; 0.86 [4.15;− 2.43]
IL-10, pg/ml, mean ± SD (IQR)	10.4 ± 7.6 (5.6–13.1)	9.3 ± 4.6 (5.8–11.9)	11.5 ± 10 (5.5–13.2)	0.456^#^; − 2.23 [2.88;− 7.35]
TNF-alpha, pg/ml, mean ± SD (IQR)	7.5 ± 0.9 (6.9–8.2)	7.6 ± 0.9 (7.2–8.2)	7.5 ± 1 (6.6–8.2)	0.838^#^; 0.13 [0.75;− 0.49]
C3, ng/ml, mean ± SD (IQR)	3626.8 ± 2322.6 (1757.7–5748.6)	3503.6 ± 2355.2 (1621–5835.8)	3750.1 ± 2410.3 (2021.9–5246.8)	0.752; − 246.52 [1321.42;− 1814.47]
Free haemoglobin, mg/ml, mean ± SD (IQR)	5 ± 4.5 (1.8–6.4)	3.8 ± 2.9 (1.9–4.6)	6.2 ± 5.6 (1.8–9.5)	0.280^#^; − 2.38 [0.56;− 5.32]
Myoglobin, ng/ml, mean ± SD (IQR)	45 ± 21.6 (24.9–59.8)	50.1 ± 23.6 (24.9–65)	40 ± 19.4 (24.9–48.5)	0.206^#^; 10.06 [24.26;− 4.14]
Haptoglobine, mg/dl, mean ± SD (IQR)	131 ± 58.2 (87.3–170.8)	116.8 ± 55.9 (74.5–158.5)	145.2 ± 59.9 (103–177)	0.139; − 28.43 [9.7;− 66.55]
Fibrinogen, mg/dl, mean ± SD (IQR)^a^	332.2 ± 77.8 (280–390)	333.3 ± 69.7 (275–377.5)	331.1 ± 88.7 (285–395)	0.931; 2.28 [55.37;− 50.81]
Syndecan-1, ng/ml, mean ± SD (IQR)	52.7 ± 54.2 (19.1–56.8)	63 ± 63.7 (20.4–62.4)	42.4 ± 43.8 (17.2–53.6)	0.313^#^; 0.36 [0.97;− 0.24]
Hyaluronan, ng/ml, mean ± SD (IQR)	132.6 ± 44.8 (107.7–142.8)	128.4 ± 30.7 (107.7–142.7)	136.7 ± 57.1 (108.7–147.9)	0.988^#^; 20.7 [56.68;− 15.29]

ASA, American Society of Anesthesiologists risk classification; BMI, body mass index; Euro Score, European System for Cardiac Operative Risk Evaluation; IL, interleukine; IQR, interquartil range; NuDesc, Nursing Delirium Screening; MD, mean difference; NYHA, New York Heart Association classification of severity of heart failure; SD, standard deviation; SOFA, sepsis-related organ failure assessment score; ARF score, acute renal failure score introduced by Thakar et al.; TNF, tumor necrosis factor

T-test, chi-square or fishers-exact test in case of n < 5

^#^Mann Whitney-U in case of not normal distribution; ^a^N = 37, control n = 18

Pre-surgical assessment revealed no significant differences in the baseline pre-surgical laboratory parameters, including creatinine, C-Reactive Protein (CRP), leukocyte count, and liver parameters (Table 1). However, slight variations were observed in pre-surgical pH levels (control group: 7.43 ± 0.04 vs. treatment group: 7.46 ± 0.04; *p* = 0.0253). Regarding pre-surgical medication intake, there were no significant differences between the groups in the use of non-steroidal anti-inflammatory drugs (NSAIDs), angiotensin-converting enzyme (ACE) blockers, angiotensin II (AT-II) antagonists, and diuretics, with the exception of statin intake, which differed between the control (n = 10/19) and treatment (n = 16/19) groups (*p* = 0.038). Analysis of pre-existing comorbidities, including nicotine and alcohol abuse, thrombosis, valve vitium, coagulopathy, pancreatitis, chronic obstructive pulmonary disease (COPD), asthma, and pulmonary embolism, revealed no significant differences between the groups. There were no differences in the occurrence of delirium or preoperative disorientation reported in patients, neither were there any significant differences in the type of cardioplegia administered during surgery, or regarding catecholamines and infused volume during surgery (supplemental Table 3).

However, a baseline difference in IL-2 levels was observed prior to surgery, with the treatment group already exhibiting higher IL-2 levels compared to the control group (control group: 8.6 ± 2 vs. treatment group: 11.4 ± 3.2 pg/ml; *p* = 0.002). No other significant differences between the groups were detected in the pre-surgical parameters assessed (Table 1), nor regarding anesthesia maintenance and surgery (supplemental Table 3).

### Primary outcome: IL-6 at ICU admission and during ICU stay

**Fig. 2:**
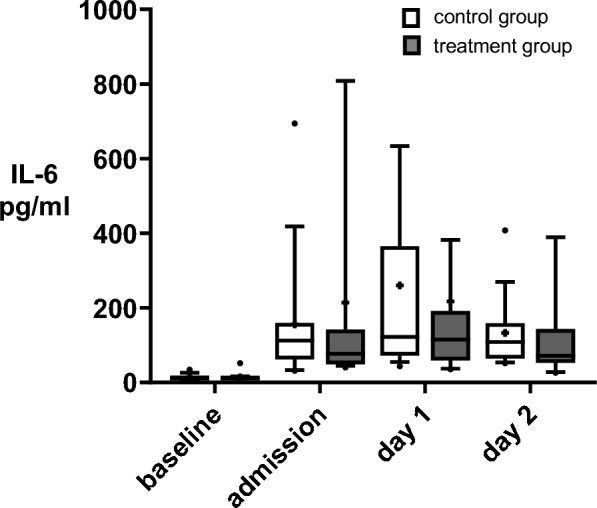
IL-6 cytokine dynamic after HA. Boxplots depicting the levels of cytokine interleukin (IL)-6 before surgery (baseline) and during the course of intensive care unit (ICU) treatment (admission, day 1 (d1), and day 2 (d2) at ICU) comparing the control group (white bars, n = 19) to the treatment group (dark grey bars, n = 19) receiving haemoadsorption (HA) during cardiothoracic surgery with cardiopulmonary bypass (CPB). Variables are presented as boxplots (10th–90th percentile, median, plus mean values)

IL-6 exhibited a notable increase at the time of ICU admission compared with baseline in both groups (1119.56% control vs 1159.41% treatment group, supplement Table 4, Fig. 2). The primary outcome, measured by IL-6 levels in pg/ml on ICU admission, did not differ (155.8 ± 159.6 pg/ml for the control group, vs 214.4 ± 328.8 pg/ml in the treatment group; *p* = 0.511). Neither on day 1 of ICU (*p* = 0.692) or on day 2 were any significant differences noted (*p* = 0.331).

### Secondary outcomes: cytokines

**Fig. 3:**
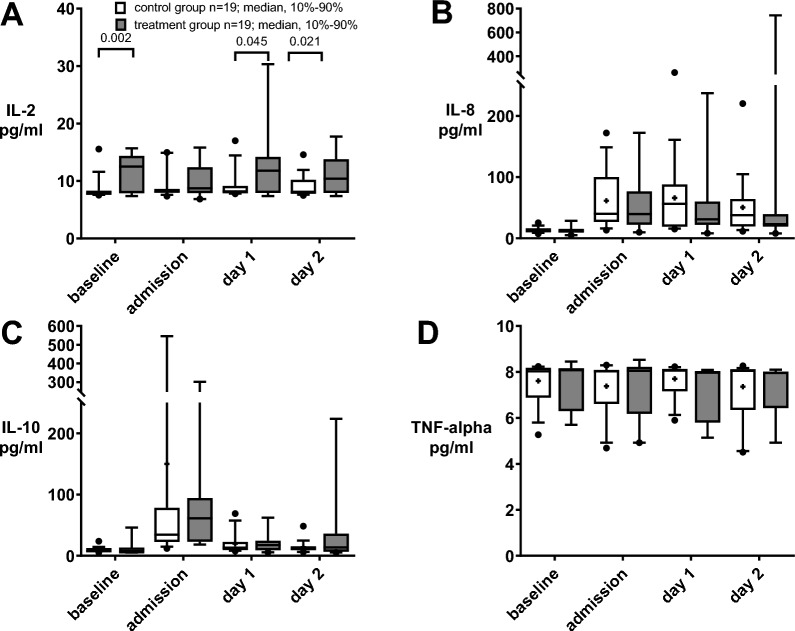
Cytokine IL-2, IL-8, IL-10, TNF-alpha dynamic after HA. Boxplots depicting the levels of cytokine IL-2 (**A**), IL-8 (**B**), IL-10 (**C**) and TNF-alpha (**D**) before surgery (baseline) and during the course of intensive care unit (ICU) treatment (admission, day 1 (d1), and day 2 (d2) at ICU) comparing the control group (white bars, n = 19) to the treatment group (dark grey bars, n = 19) receiving haemoadsorption (HA) during cardiothoracic surgery with cardiopulmonary bypass (CPB). Variables are presented as boxplots (10th–90th percentile, median, plus mean values). Statistical analysis: Mann–Whitney-U-test of raw data after normality testing (Kolmogorov–Smirnov). *p < 0.05. N = 38

At ICU admission, no significant differences were detected for post-surgical cytokine levels in the patient’s blood between the control and treatment groups (supplement Table 4, Fig. 3A–D). In the course of the further intensive care treatment (d1, d2 and follow-ups) no differences regarding cytokines were detected with one exception: Comparable to before surgery, low levels of IL-2 were detected for the control group on d1 (control group: 9.2 ± 2.5 vs treatment group: 12.1 ± 5.4 pg/ml; *p* = 0.045) and d2 (control group: 9 ± 1.9 vs treatment group: 11.1 ± 3.2 pg/ml; *p* = 0.021, Fig. 3A). However, over the course of ICU days, IL-10 difference was higher in patients who received the treatment.

Given the baseline differences observed for IL-2, an exploratory approach was employed subtracting cytokine levels relative to the previous time point. Conversely, IL-2 decreased significantly in the treatment group post-surgery at the time of ICU admission compared to baseline (*p* = 0.016). Fluctuations in IL-2 clearance and secretion between time points were moderate, ranging from a cytokine reduction of − 9.02% in the treatment group to a cytokine increase of 7.13% in the control group. Whereas IL-10 substantially increased in both groups (2209.08% control vs. 924.37% treatment), with a significant difference between groups on day 2 (Supplement Fig. 1). Whereas IL-8 exhibited a moderate increase in cytokine levels post-surgery.

**Fig. 4:**
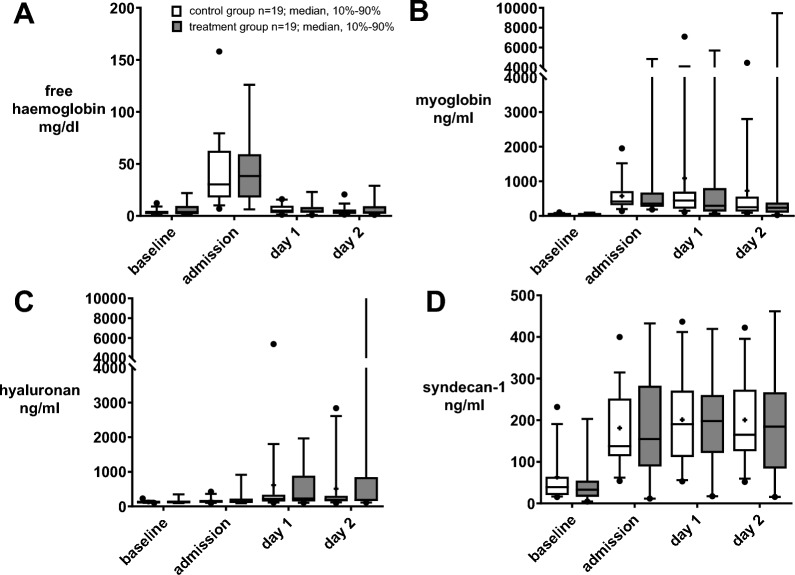
Plasma protein dynamic after HA. Boxplots depicting the levels of free haemoglobin (**A**), myoglobin (**B**), hyaluronan (**C**) and syndecan (**D**) before surgery (baseline) and during the course of intensive care unit (ICU) treatment (admission, day 1 (d1), and day 2 (d2) at ICU) comparing the control group (white bars, n = 19) to the treatment group (dark grey bars, n = 19) receiving haemoadsorption (HA) during cardiothoracic surgery with cardiopulmonary bypass (CPB). Variables are presented as boxplots (10th–90th percentile, median, plus mean values). Statistical analysis: Mann–Whitney-U-test of raw data after normality testing (Kolmogorov–Smirnov). *p < 0.05. N = 38

During the ICU stay, free haemoglobin, myoglobin, hyaluronan and syndecan-1 did not differ significantly between the study groups (Fig. 4, supplement Table 4).

At admission to ICU the treatment group showed a slight decrease in albumin (control group: 2.5 ± 0.5 vs treatment group: 2.2 ± 0.4 g/dl; *p* = 0.028). Lower heparan sulfate was observed at admission (control group: 2025.8 ± 1358.6 vs treatment group: 1196.9 ± 646.9 ng/ml; *p* = 0.024). Albumin levels did not recover (day 1: control group: 2.8 ± 0.4 vs. treatment group: 2.5 ± 0.5 g/dl, *p* = 0.02; day 2: control group: 2.9 ± 0.5 vs. treatment group: 2.6 ± 0.3 g/dl, *p* = 0.027).

### Clinical outcome parameters

**Fig. 5:**
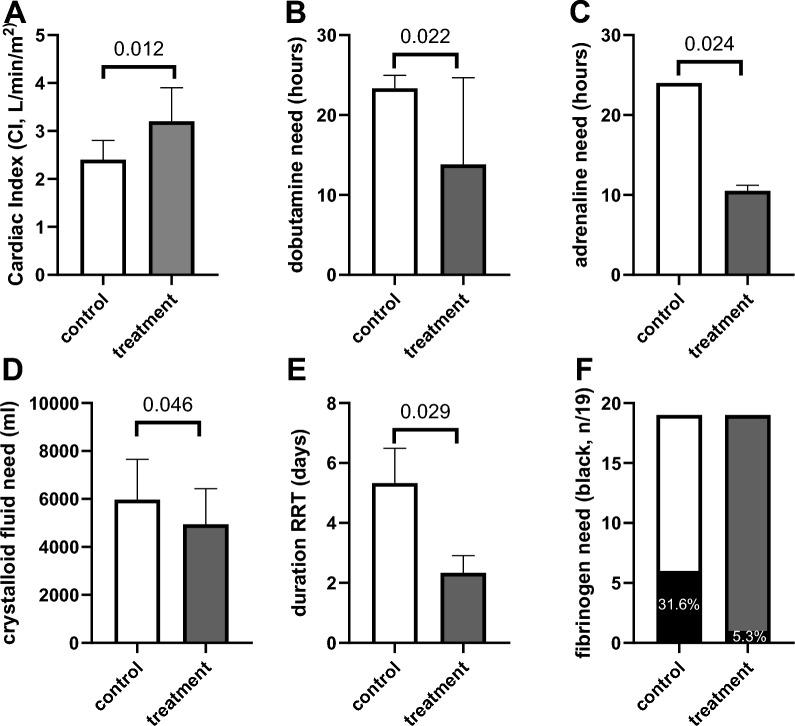
Clinical outcomes after HA. Bar graphs depicting Cardiac Index (CI) (**A**), duration of dobutamine need in hours until d2 (control: n = 6, treatment group n = 10) (**B**), duration of adrenaline need in hours until d3 (control: n = 2, treatment group n = 2) (**C**), need for crystalloid fluid replacement up to d1 (**D**), duration of renal-replacement-therapy (RRT) (control: n = 3, treatment group n = 3) (**E**), number of patients needing fibrinogen (black) (**F**) during the course of intensive care unit (ICU) treatment (admission, day 1 (d1), and day 2 (d2) at ICU) comparing controls (white bars, n = 19) and treatment group (dark grey bars) having received haemoadsorption (HA) during cardiothoracic surgery with cardiopulmonary bypass (CPB). All variables are presented as mean ± standard deviation (SD), except panel F presenting number of patients and percentage. N = 39. Statistical analysis: **A**–**C** t-test, **D** chi-square

Regarding haemodynamic stability (supplement Table 4) recovery of the Cardiac Index (CI), with increased CI in the treatment group on day 2 was observeved (day 1: N = 28, 2.8 ± 0.7 vs. 2.7 ± 0.5 l/min/m^2^, *p* = 0.012; day 2: N = 15 3.2 ± 0.7 vs. 2.4 ± 0.4 l/min/m^2^, *p* = 0.012, Fig. 5A, supplement Table 4). Similarly, a significantly shorter duration of dobutamine support (control group: 23.3 ± 1.6 vs treatment group: 13.8 ± 10.9 h; n = 16, *p* = 0.022) up to day 2 was observed. The need for adrenaline up to day 3 was shorter in the HA group (control group: 24 ± 0 vs treatment group: 10.5 ± 0.7 h; n = 4, p = 0.024) (Fig. 5B, C). Though, noradrenaline need did not differ between groups (n = 38). Throughout this period, the requirement for crystalloid fluid was lower in the treatment group (control group: 5967.1 ± 1690.1 vs. treatment group: 4942.6 ± 1489 ml; *p* = 0.046) (Fig. 5D).

No other significant differences regarding clinical outcome parameters, including daily SOFA scores, time on mechanical ventilation, incidence of postoperative delirium, infectious complications, antibiotics, were detected (supplement Table 4, supplement Table 5). There were also no differences in secondary laboratory outcomes observed in blood parameters except for significant differences in alanine transaminase (ALT) levels between the control group (33.7 ± 35.8 U/L) and the treatment group (27.1 ± 34.4 U/L; *p* = 0.0484#). Regarding the number of secondary adverse events no significant difference was detected (supplemental Table 5).

In each study group, three patients required renal replacement therapy, and a shorter duration of required renal replacement therapy was observed in the treatment group (control group: 5.3 ± 1.2 vs. treatment group: 2.3 ± 0.6 days; n = 6, *p* = 0.029, Fig. 5E) was observed. At admission no increased need for transfusion, substitution of coagulation factors, nor albumin by the blinded intensive care team were observed.

Interestingly, regarding the need for transfusion and coagulation factors, the need for fibrinogen was even significantly lower on d1 in the treatment group (control group: 1.1 ± 1.7, n = 6 vs. treatment group: 0.4 ± 1.8 g; n = 1, *p* = 0.036 Fig. 5F).

### Follow-up

Regarding follow-up (supplement Table 5), the mean length of stay was comparable across groups, with no statistically significant differences observed in either the ICU or hospital length of stay (*p* = 0.87 and *p* = 0.484, respectively). Readmission rates to the ICU were identical among groups.

### Intraoperative haemoadsorption

**Fig.6:**
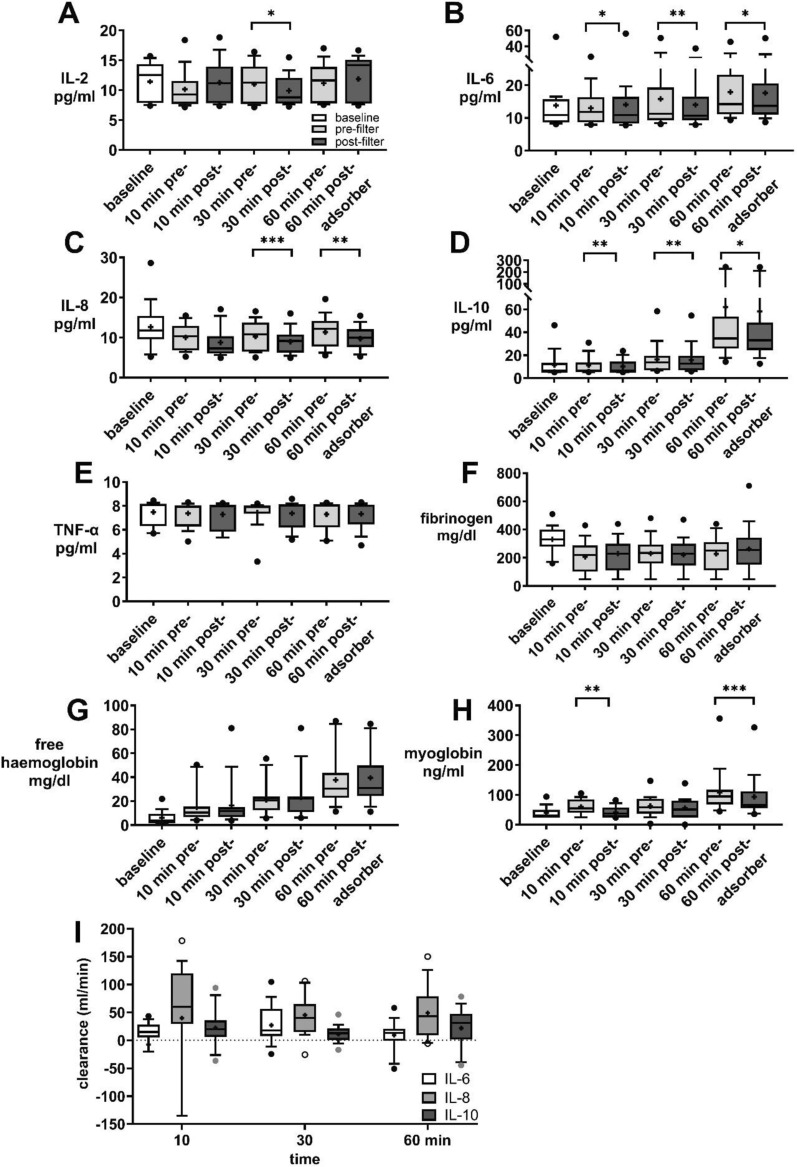
Pre- versus post-adsorber cytokine serum concentrations under HA during surgery and clearance. Boxplots illustrating the levels of cytokines, IL-6 (**B**), IL-8 (**C**), IL-10 (**D**), TNF-alpha (**E**)), fibrinogen (**F**), free haemoglobin (**G**), and myoglobin (**H**) pre- and post-adsorber (dark grey) haemoadsorption (HA) during cardiothoracic surgery with cardiopulmonary bypass (CPB) at 10, 30, and 60 min compared to baseline (preoperative, white). (**I**) Clearance of IL-6, IL-8, and IL-10 at 10, 30, and 60 min after CPB initiation. Individual clearance trajectories demonstrate positive clearance values for IL-6 and IL-8 at least at one timepoint. For IL-6, three patients, for IL-8, two patients, initially exhibited negative clearance values. Data is based on n = 19 participants from the treatment group only. All variables are presented as boxplots (10th–90th percentile, median, plus mean values). Statistical analysis for (**A**–**H**) Wilcoxon test if normality was not met (Kolmogorov–Smirnov) or paired t-test for fibrinogen and TNF-α. n = 19, *p < 0.05, ** < 0.01, *** < 0.001

During surgery, significant pre- and post-adsorber differences were observed in the levels of IL-2, IL-6, IL-8, and IL-10 between pre- and post-adsorber stages during surgery. Lower values for these cytokines were consistently measured post-adsorber at 30 and 60 min into the cardiopulmonary bypass time, and for some cytokines (IL-6, IL-10 and myoglobin) this occurred even after 10 min with a measurable clearance of cytokines (Fig. 6).

The concentrations of myoglobin and heparan sulfate were significantly reduced post-adsorber compared to pre adsorber levels (10 min: pre: 686.7 ± 492.8 vs. post: 398.3 ± 435.3 mg/dl; *p* = 0.01, 30 min: pre: 631.3 ± 474.1 vs. post: 412.3 ± 307.6 mg/dl; *p* < 0.001, 60 min: pre: 657 ± 527.9 vs. post: 515.9 ± 386.2 mg/dl, *p* = 0.036). However, no significant differences were detected for TNF-α, fibrinogen, free haemoglobin, syndecan-1, C3a, haptoglobin, and hyaluronan. No adverse events were observed (supplement Table 4).

## Discussion

In this study, our primary objective was to determine whether intraoperative HA during elective on-pump cardiac surgery leads to reduced postoperative blood cytokine levels (that is, IL-6). In this blinded randomised controlled trial IL-6 levels did not differ between the study groups on ICU admission, nor on day 1 and 2 in the ICU. We proved that utilising HA leads to a reduction in cytokine levels of IL-2, IL-6, IL-8, and IL-10 post-adsorber levels. However, this reduction was not consistently observed in the patients’ serum samples. Although there were indications of minor improvements in haemodynamic stability, no direct causation can be established. We were able to detect some effects on post-surgical cytokines, in contrast to previous studies that did not detect any relevant cytokine removal [20, 34]. In our study population, IL-6 levels did not differ between groups with and without HA treatment. Similarly, several studies were not able to demonstrate significant reductions in postoperative IL-6 levels in cardiac surgery with HA [35–37]. This raises the question of whether IL-6, as a component of the inflammatory cascade, should be the sole parameter monitored intra- and post-operatively. Variations in results regarding cytokine secretion, especially IL-6 and TNF-α, and consequently elimination by HA during cardiac surgery with CPB, may also be attributed to previously described inter-individual differences and genetic polymorphisms [38]. It is now well established that IL-6 exhibits significant variability in kinetics among individuals and may not be released in adequate quantities during elective heart surgery involving the use of a heart–lung machine [39]. Notably, there are indications that inflammation persists beyond the termination of CPB, as proinflammatory cytokine levels were comparatively lower in the treatment group than in the control group, which could account for the differing cytokine levels on days 1 and 2 in the ICU. Cytokines are known to be secreted at the end or immediately after surgery. The treatment time of HA correlates with the length of CPB, whereas the liberation of cytokines is reported to peak during CPB and 6 h thereafter [12]. The recommended time for HA is up to 72 h and cytokine elimination is dependent on substance gradient [40, 41]. Moreover, although the patients exhibited severe illness indicated by elevated EuroSCOREs, the initial inflammatory response triggered by the surgical procedure or underlying disease might not have been of sufficient magnitude to elicit a significant cytokine release of all cytokines similar to other studies [20] and in contrast to massive cytokine release during cardiac surgeries of patients with endocarditis [19]. Thus, the long-term effects of HA during surgery on ICU treatment may not have been discernibly influenced by this factor. It is indeed possible that the inflammatory burden in our patient population was not severe enough to demonstrate a substantial impact of CytoSorb on IL-6 levels, as the levels remained relatively low. This raises an important consideration about whether extracorporeal adsorption might be more beneficial in patients with a more pronounced inflammatory response or in those undergoing more complex surgeries with higher risk of hyperinflammation. Fittingly a recent randomized controlled trial demonstrated, that perioperative adsorber treatment was particularly effective in high-risk subgroups, including older patients, with chronic kidney disease, diabetes, hypertension and reduced left ventricular ejection fraction [42].

Alternatively, post-surgical cytokine release, even quite strong for some cytokines may have predominantly occurred following HA. In our study, the majority of patients in the treatment group exhibited positive clearance values for all cytokines across multiple time points. However, some patients showed initial or temporary negative clearance values. Similar findings have been reported in drug studies, where negative clearance values were observed, especially at concentrations measured directly on the adsorber. Potential explanations include cellular release, desorption, a re-release from polymer beads, and adsorber saturation. Competition for binding sites and displacement by higher concentrations of competing proteins are also possible contributing factors. [43–45]

The dynamics of IL-10, an anti-inflammatory cytokine, are known to counteract hyperinflammation [46–48]. In our investigation, we observed elevated IL-10 levels in the HA treatment group during the ICU stay, aligning with the findings of other researchers suggesting a prolonged anti-inflammatory effect of IL-10 mediated by HA adsorption [12]. These indications should be further evaluated for consistency in future investigations.

As previously reported, regarding measurements before and after the haemoadsorption, pre/post-adsorber, heparan sulfate was efficiently adsorbed both before and after the adsorber, showing significantly lower levels in the treatment group. Consequently we verified that this soluble component of the endothelial glycocalyx, recognized as exacerbating an ongoing inflammatory response, was effectively removed through haemoadsorption [49]. However, other important glycocalyx components like syndecan-1 and hyaluronan could not be effectively reduced making a general benefit on the endothelial surface lining questionable. In line with our results of reduced post vs. pre adsorber cytokine levels and a clearance by HA during CBP of all cytokines, except TNF-alpha, other authors have documented decreases in measured proinflammatory cytokine concentrations after HA therapy in cardiac surgery, but with no [12] or only transient effects on postoperative outcome, e.g. on haemodynamic stability [37], indicating its effectiveness in mitigating the inflammatory reaction [12, 37].

Our results indicate cytokine elimination pre versus post-adsorber. However, the observed minor significant differences in cytokine levels at ICU, and only mild favorable clinical outcomes may be attributed to insufficient HA therapy duration.

So far studies have been described as low quality and inconclusive, and have not shown patient-relevant endpoints or effects on organ dysfunction after HA in cardiac surgery [34]. There is contrasting evidence regarding improvement of postoperative organ failure by HA [11, 13, 50].

In our prospective RCT hints towards improved haemodynamic stability were observed, with increased cardiac index following HA treatment. A reduction in the need for inotropes was observed in the treatment group, consistent with transient effects from other RCTs [37] and results from observational studies [41, 51, 52]. Whether the observed improvements are caused by the HA device cannot be determined, warranting further investigations in larger randomized studies with clinical endpoints.

In favour of HA, no reduction in plasma proteins or coagulation factors were detected post-adsorber during surgery. Previously described signs of coagulation activation were not observed [20]. Indeed, in our study at ICU admission the treatment group exhibited decreased albumin. However, those reductions were presumably without clinical relevance, since the parameters did not lead to increased rates of transfusion, substitution of coagulation factors, or albumin substitution by the blinded ICU team. Accordingly, the average removal of albumin over an extended period (7 days) was found to be less than 10% after HA therapy, deemed clinically not relevant [53].

These findings collectively suggest that the adsorption process may lead to the reduction of specific cytokines, without causing undesirable effects on coagulation factors or necessitating additional transfusions. Importantly, no adverse events related to the use of HA were detected in our study, and any complications observed in this cohort were not associated with HA, as previously described [20].

The secondary aims of our study were to explore effects on mechanisms of postoperative organ failure, since it is known that particularly elevated levels of free haemoglobin and myoglobin contribute to the pathogenesis of cardiac surgery-associated organ failure [54]. We did indeed observe removal of myoglobin pre- vs. post-adsorber. However, effects on postoperative plasma proteins were not detected. In favor of the post-surgical kidney function, our study demonstrated a significant reduction of the need for RRT although in an altogether small number of patients. In line with the described trends, in the recent randomized controlled SIRAKI02 trial, connecting a nonselective extracorporeal blood purification membrane to the continuous kidney replacement therapy during CPB, significantly reduced the incidence of acute kidney injury over seven days [42]. For other aspects there is contrasting evidence regarding improvement of postoperative organ failure by HA [11, 13, 50]. Comparable to our results, a retrospective investigation showed a significant decrease in SOFA score as an surrogate parameter for organ failure [55].

In our study an overall balanced group distribution was achieved by randomisation, as evidenced by pre-surgical assessments not revealing differences in baseline laboratory parameters and medication intake, only the differences in baseline IL-2 values were higher in the treatment group without evident reason which lasted throughout the whole post-surgical observation period. Only few pre-surgical dropouts could have influenced baseline characteristics, however, no dropouts occurred after application of treatment.

### Limitations

The small sample size of our study may limit the generalizability of the findings and reduce statistical power, potentially preventing some observed effects from reaching statistical significance. While the sample size calculation was appropriate for the primary outcome, and other studies with similar designs have successfully reached their endpoints, the significant secondary outcomes should be considered exploratory, therefore no statistical correction accounting for multiple testing was conducted, but the initial sample size was not calculated for those outcomes and may therefore be underpowered for these outcomes. This may weaken the robustness of the results, particularly regarding pre-post cytokine levels, which showed considerable variability and inconsistent cytokine concentrations over time, with only select time points showing significant differences.

Furthermore, although a specialized heart–lung machine setup, including an additional pump, was employed to ensure constant blood flow and optimize the adsorber’s functionality, it cannot be entirely ruled out that the circuit may not have removed the majority of cytokines. The minor significant differences observed in cytokine levels at the ICU and the modest clinical benefits may be due to insufficient haemoadsorption therapy duration or the treatment's applicability to a population with a more substantial inflammatory burden [56]. The, albeit marginal, hints regarding catecholamines in our study are also shown in other studies. Further research in this direction is therefore recommended. In future studies, a clinical endpoint should be selected. A potentially suitable and now well-validated parameter that also correlates with the clinical outcome of patients could be for example the Vasoactive Inotropic Score [57, 58].

## Conclusion

Taken together, our study suggests, that the use of HA did not significantly reduce IL-6 in patients undergoing cardiac surgery. We did show that cytokine levels post-adsorber were reduced, but no lasting effects were detected on ICU. Improved haemodynamic stability was noted without notable adverse effects. Further research, including large-scale randomised controlled trials is needed to elucidate the optimal timing, dosing, and patient selection criteria for HA therapy in this context.

## Supplementary Information

Supplementary Material 1.

Supplementary Material 2.

Supplementary Material 3.

Supplementary Material 4.

Supplementary Material 5.

Supplementary Material 6.

## Data Availability

After publication, data will be made available to other investigators upon reasonable requests to the corresponding author.

## Abbreviations

ACE: Angiotensin-converting enzyme
ACT: Activated clotting time
AIDS: Acquired immune deficiency syndrome
AKI: Acute kidney injury
AKIN: Acute kidney injury network
ALT: Alanine aminotransferase
ASA: American Society of Anesthesiologists
AT-II: Angiotensin II
C3a: Complement component 3a
CD4: Cluster of differentiation 4
CE: Conformité Européenne
CI: Cardiac Index
CONSORT: Consolidated Standards of Reporting Trials
COPD: Chronic obstructive pulmonary disease
CPB: Cardiopulmonary bypass
CRF: Case Report Form
CRP: C-reactive protein
CVVHD: Continuous veno-venous haemodialysis
CVVHDF: Continuous veno-venous haemodiafiltration
D1/D2: Day one or two after surgery
DO2I: Oxygen Delivery Index
DRKS: German Register for Clinical Studies
EuroSCORE: European System for Cardiac Operative Risk Evaluation
EVLWI: Extravascular Lung Water Index
GFR: Glomerular filtration rate
GEDI: Global End-Diastolic Volume Index
HA: Haemoadsorption device, CytoSorb®
HLM: Heart–lung machine
ICH-GCP: International Conference on Harmonisation-Good Clinical Practice
ICU: Intensive care unit
IL: Interleukin
IQR: Interquartile range
ISO: International Organisation for Standardisation
KDIGO: Kidney Disease Improving Global Outcomes
NSAID: Non-steroidal anti-inflammatory drug
NuDESC: Nursing Delirium Screening Scale
PCT: Procalcitonin
RCT: Randomised controlled trial
REDCap: Research electronic data capture
RRT: Renal replacement therapy
SD: Standard deviation
SIRS: Systemic inflammatory response syndrome
SOFA: Sequential organ failure assessment
TLR-4: Toll-like receptor 4
TNF-alpha: Tumor necrosis factor alpha
