# Supplementary material for: REmoval of cytokines during CArdiac surgery (RECCAS): a randomised controlled trial
Source: Crit Care. 2024 Dec 12;28:406. doi: 10.1186/s13054-024-05175-9 (PMC11639119; doi:10.1186/s13054-024-05175-9)
Supplement: Supplementary file 1 — Supplementary Material 1. [file 13054_2024_5175_MOESM1_ESM.docx]

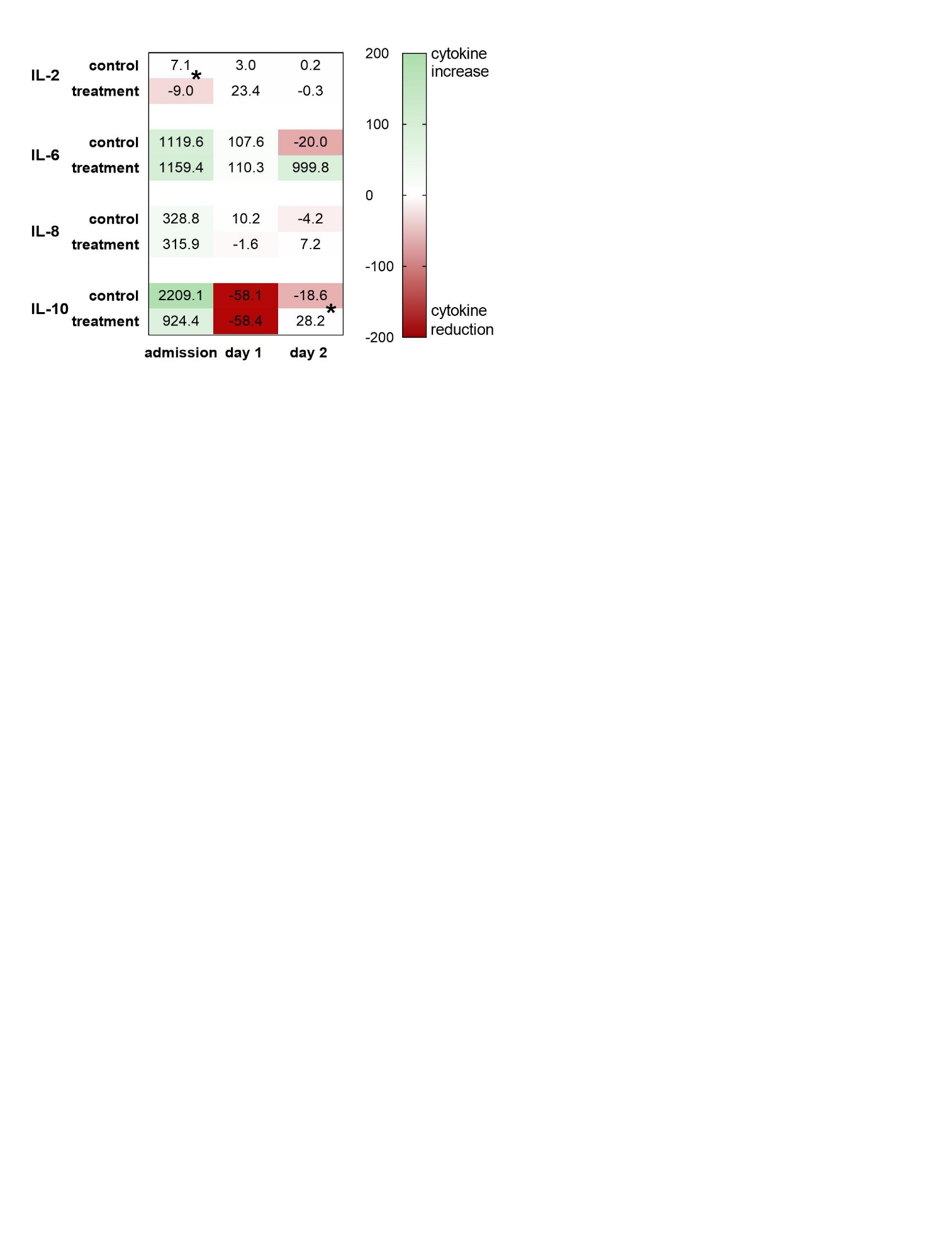


**Supplement figure 1**

Heatmap of cytokine difference subtracted from previous timepoint of each patient. Data are means of percentage of change subdivided into control and treatment group for cytokines IL-2, IL-6, IL-8, IL-10. Values highlighted red show a reduction of cytokines, whereas shades of green signal different levels of elevation of cytokine levels.

Statistical analysis: Mann-Whitney-U-test of raw data after normality testing (Kolmogorov-Smirnov). * p < 0.05. N = 38.

Abbreviations: CPB: Cardiopulmonary Bypass, d1: day 1 of ICU treatment, d2: day 2 of ICU treatment HA: Hemoadsorption, ICU: Intensive Care Unit, IL-2: Interleukin-2, IL-6: Interleukin-6, IL-8: Interleukin-8, IL-10: Interleukin-10.
