# Supplementary material for: REmoval of cytokines during CArdiac surgery (RECCAS): a randomised controlled trial
Source: Crit Care. 2024 Dec 12;28:406. doi: 10.1186/s13054-024-05175-9 (PMC11639119; doi:10.1186/s13054-024-05175-9)
Supplement: Supplementary file 3 — Supplementary Material 3. [file 13054_2024_5175_MOESM3_ESM.docx]

**Supplemental table 2 Summary of outcome parameters at different time points**

|  |  |  | pre/post-adsorber- during CBP^a^ | | |  |  | |  | |  | |  | |  |  |
| --- | --- | --- | --- | --- | --- | --- | --- | --- | --- | --- | --- | --- | --- | --- | --- | --- |
|  | Hospital admission | Baseline | 10 min | 30 min | 60 min | ICU admission | | ICU d 1 | | ICU d 2 | | ICU d 3-28 | | Follow-up (hospital discharge) | |  |
|  |  |  |  |  |  |  | |  | |  | |  | |  | |  |
| **Cytokines** |  | **X** | **X** | **X** | **X** | **X** | | **X** | | **X** | |  | |  | |  |
| IL-6 (primary outcome) |  | X | X | X | X | X | | X | | X | |  | |  | |  |
| IL-2 |  | X | X | X | X | X | | X | | X | |  | |  | |  |
| IL-8 |  | X | X | X | X | X | | X | | X | |  | |  | |  |
| IL-10 |  | X | X | X | X | X | | X | | X | |  | |  | |  |
| TNF-alpha |  | X | X | X | X | X | | X | | X | |  | |  | |  |
| **Plasma proteins** |  | **X** | **X** | **X** | **X** | **X** | | **X** | | **X** | |  | |  | |  |
| C3a | |  | X | X | X | X | X | | X | | X | |  | |  | |
| Free haemoglobin | |  | X | X | X | X | X | | X | | X | |  | |  | |
| Haptoglobin | |  | X | X | X | X | X | | X | | X | |  | |  | |
| Myoglobin | |  | X | X | X | X | X | | X | | X | |  | |  | |
| Fibrinogen | |  | X | X | X | X | X | | X | | X | |  | |  | |
| Syndecan-1 | |  | X | X | X | X | X | | X | | X | |  | |  | |
| Hyaluronan | |  | X | X | X | X | X | | X | | X | |  | |  | |
| Heparan sulphate | |  | X | X | X | X | X | | X | | X | |  | |  | |
| **Blood gas analysis** | **X** | **X** | **X** | **X** | **X** | **X** | | **X** | | **X** | | **X** | | **X** | |  |
| **Routine blood samples** | **X** | **X** |  |  |  | **X** | | **X** | | **X** | | **X** | | **X** | |  |
| **Clinical outcome parameters** |  |  |  |  |  | **X** | | **X** | | **X** | | **X** | | **X** | |  |
| SOFA scores |  |  |  |  |  | X | | X | | X | | X | |  | |  |
| haemodynamics and cardiac output |  |  |  |  |  | X | | X | | X | | X | |  | |  |
| mechanical ventilation |  |  |  |  |  |  | | X | | X | | X | |  | |  |
| extracorporeal haemodynamic support |  |  |  |  |  |  | | X | | X | | X | |  | |  |
| postoperative delirium |  |  |  |  |  |  | | X | | X | | X | |  | |  |
| infectious complications, antibiotics |  |  |  |  |  |  | | X | | X | | X | |  | |  |
| fluid balance |  |  |  |  |  |  | | X | | X | | X | |  | |  |
| transfusion or coagulation factors |  |  | X | X | X | X | | X | | X | | X | |  | |  |
| renal function |  |  |  |  |  |  | | X | | X | | X | | X | |  |

^a^lntraoperative “in vitro” cytokine kinetics and markers of secondary objectives were only assessed in patients allocated to the intervention group with haemoadsorption device at 10, 30 and 60 min after initiation of cardiopulmonary bypass (CPB). For this, blood samples at the time points were drawn from the CPB circuit both before and after the haemoadsorption device (pre-/adsorber). The secondary outcomes included baseline presurgical laboratory parameters (creatinine, C-reactive protein (CRP), and liver parameters (Alanine Aminotransferase (ALT)), medication, diseases, parameters of anaesthesia & surgery duration of surgery, CPB and aortic crossclamping, duration per 24 hours and total cumulative duration and number of patients needing vasopressors and inotropics, amount of fluids and fluid balance, urine output, need of transfusion and coagulation factors, cytokine serum concentrations (IL-2, IL6, IL-8, IL-10, TNF-α) and plasma proteins (C3a, haptoglobin, fibrinogen, syndecan-1, hyaluronan, heparan sulfate, free haemoglobin and myoglobin) at different timepoints (baseline, during surgery, ICU admission, d1 and d2), blood gas analysis and laboratory parameters (pH, pO2, pCO2, haemoglobin, lactate, bicarbonate, base excess, glucose, Procalcitonin (PCT)), additionally at ICU daily clinical outcomes and signs of postoperative organ failure every 24 hours for seven days or until patients were discharged from ICU were collected, SOFA scores, postoperative delirium, changes in haemodynamics and cardiac output ,Cardiac Index (CI), Global End-Diastolic Volume Index (GEDI), Extravascular Lung Water Index (EVLWI), duration of mechanical ventilation, infectious complications, renal function (creatinine, Glomerular Filtration Rate (GFR)), renal replacement therapy, need of transfusion or coagulation factors, secondary complications. A follow-up at hospital discharge evaluated length of stay at ICU, length of stay in the hospital, renal function on ICU/hospital discharge and mortality.
