# Supplementary material for: REmoval of cytokines during CArdiac surgery (RECCAS): a randomised controlled trial
Source: Crit Care. 2024 Dec 12;28:406. doi: 10.1186/s13054-024-05175-9 (PMC11639119; doi:10.1186/s13054-024-05175-9)
Supplement: Supplementary file 4 — Supplementary Material 4. [file 13054_2024_5175_MOESM4_ESM.docx]

|  | **Total** | **control** | **treatment** | **p-value; MD [CI]** |
| --- | --- | --- | --- | --- |
| **Number (n)** | **38** | **19** | **19** |  |
| noradrenaline, cumulative, µg, mean ± SD (IQR) | 3724.6 ± 4330.3  (1260.1 - 5252.4) | 3485.9 ± 3225.5  (1304.8 - 4422.3) | 3963.2 ± 5391.1  (1347.8 - 5311.3) | 0.743; -477.23  [2445.77;-3400.23] |
| adrenaline, cumulative, µg, mean ± SD (IQR) | 348.4 ± 1598.7 (0 - 0) | 666.3 ± 2272.6 (0 - 0) | 30.5 ± 133 (0 - 0) | 0.143^#^; 635.83  [1695.04;-423.39] |
| number of patients needing, n (%) |  |  |  |  |
| adrenaline | 5 (13.2) | 4 (10.5) | 1 (2.6) | 0.15 |
| dobutamine | 37 (97.4) | 18 (47.4) | 19 (50) | 0.311 |
| terlipressin | 4 (10.5) | 2 (5.3) | 2 (5.3) | 1 |
| milrinon | 25 (65.8) | 12 (31.6) | 13 (34.2) | 0.732 |
| levosimendan | 1 (2.6) | 1 (2.6) | 0 (0) | 0.311 |
| infused volume, ml, mean ± SD (IQR) | 2296.1 ± 673.4  (2000 - 2500) | 2421.1 ± 754.7  (1750 - 3000) | 2171.1 ± 595.7  (2000 - 2500) | 0.265; 250  [697.33;-197.33] |
| urine output, ml, mean ± SD (IQR) | 1221.1 ± 987.2  (597.5 - 1650) | 1136.3 ± 926.9  (420 - 1465) | 1305.8 ± 1087.8  (700 - 1650) | 0.397^#^; -169.53  [495.4;-834.46] |

**Supplemental table 3: during anesthesia & surgery**
