# Supplementary material for: REmoval of cytokines during CArdiac surgery (RECCAS): a randomised controlled trial
Source: Crit Care. 2024 Dec 12;28:406. doi: 10.1186/s13054-024-05175-9 (PMC11639119; doi:10.1186/s13054-024-05175-9)
Supplement: Supplementary file 5 — Supplementary Material 5. [file 13054_2024_5175_MOESM5_ESM.docx]

Supplemental table 4: Outcomes ICU admission and following days, primary and secondary outcomes and follow-up and total cumulative outcomes of ICU therapy N=38

|  | **Total** | **control** | **treatment** | **p-value; MD [CI]** |
| --- | --- | --- | --- | --- |
| **Number (n)** | **38** | **19** | **19** |  |
| **primary outcome** |  |  |  |  |
| IL-6, pg/ml, mean ± SD (IQR) | 185.1 ± 253.3  (57.5 - 153.8) | 155.8 ± 159.6  (64.9 - 158.4) | 214.4 ± 328.8  (52.6 - 118) | 0.511^#^; -58.53  [111.53;-228.59] |
| IL-6, pg/ml, mean ± SD (IQR) | 238.8 ± 322.2  (72.4 - 267.9) | 260.3 ± 282  (76.8 - 317.7) | 217.4 ± 372.4  (65.8 - 184.1) | 0.692; 42.84  [260.2;-174.52] |
| IL-6, pg/ml, mean ± SD (IQR) | 8786.5 ± 52719.7  (57.2 - 151.8) | 133.2 ± 93  (66.2 - 156.6) | 17439.8 ± 75560.9  (54.8 - 135.9) | 0.331; -17306.56  [17850.21;-52463.34] |
|  |  |  |  |  |
| **Secondary outcomes** |  |  |  |  |
| IlL-2, pg/ml, mean ± SD (IQR) | 9.6 ± 2.5 (8 - 11.3) | 9 ± 2.3 (8 - 8.5) | 10.1 ± 2.7 (7.9 - 12.2) | 0.321^#^; -1.09 [0.54;-2.73] |
| IlL-2, pg/ml, mean ± SD (IQR) | 10.7 ± 4.4 (7.9 - 12.9) | 9.2 ± 2.5 (7.9 - 9.1) | 12.1 ± 5.4 (8 - 14) | 0.045; -2.89 [-0.11;-5.66] |
| IlL-2, pg/ml, mean ± SD (IQR) | 10.1 ± 2.8 (7.9 - 11.8) | 9 ± 1.9 (7.8 - 10.1) | 11.1 ± 3.2 (8 - 13.3) | 0.021; -2.1 [-0.36;-3.83] |
| IlL-8, pg/ml, mean ± SD (IQR) | 57.4 ± 44.7 (26.5 - 84.9) | 61.4 ± 47.8 (27.4 - 93.8) | 53.4 ± 43.5 (22.7 - 67.9) | 0.589^#^; 8.04 [38.13;-22.04] |
| IlL-8, pg/ml, mean ± SD (IQR) | 56.1 ± 57.1 (20.4 - 60.1) | 66.1 ± 63.6 (19.5 - 81.1) | 46 ± 51.1 (22.1 - 53.5) | 0.29; 20.11 [58.08;-17.87] |
| IlL-8, pg/ml, mean ± SD (IQR) | 60.4 ± 118.6 (19.4 - 56.1) | 50.4 ± 48 (19.5 - 63) | 70.3 ± 164.9 (19.7 - 38) | 0.62; -19.82 [60.06;-99.71] |
| IlL-10, pg/ml, mean ± SD (IQR) | 111.5 ± 205 (23.3 - 87.4) | 150.1 ± 284.6 (23.1 - 76.4) | 73 ± 67.6 (25.3 - 91.2) | 0.589^#^; 77.2 [213.29;-58.9] |
| IlL-10, pg/ml, mean ± SD (IQR) | 20.3 ± 15.4 (9.3 - 23.4) | 19.7 ± 17 (9.3 - 19.8) | 20.9 ± 14.6 (10.7 - 24.1) | 0.807; -1.26 [9.17;-11.7] |
| IlL-10, pg/ml, mean ± SD (IQR) | 24 ± 39.1 (6.9 - 15.3) | 14 ± 9.5 (9.3 - 14.9) | 34 ± 54.1 (6.6 - 25.4) | 0.129; -20 [5.57;-45.57] |
| TNF-alpha, pg/ml, mean ± SD (IQR) | 7.4 ± 1.1 (6.7 - 8.2) | 7.4 ± 1.2 (6.7 - 8.1) | 7.4 ± 1.1 (6.6 - 8.2) | 0.483^#^; -0.06 [0.71;-0.82] |
| TNF-alpha, pg/ml, mean ± SD (IQR) | 7.4 ± 1 (7 - 8.1) | 7.7 ± 0.7 (7.4 - 8.1) | 7.2 ± 1.2 (5.9 - 8) | 0.094; 0.55 [1.19;-0.09] |
| TNF-alpha, pg/ml, mean ± SD (IQR) | 7.3 ± 1.2 (6.5 - 8.1) | 7.4 ± 1.2 (6.7 - 8.1) | 7.3 ± 1.2 (6.5 - 8) | 0.777; 0.11 [0.9;-0.68] |
| C3a, ng/ml, mean ± SD (IQR) | 2961.2 ± 2636.6  (988.3 - 4688.2) | 3000.7 ± 3360.8  (886.3 - 4072.2) | 2921.7 ± 1837.8  (1396.6 - 4994) | 0.373^#^; 78.94  [1861.18;-1703.3] |
| C3a, ng/ml, mean ± SD (IQR) | 4016.1 ± 3148.1  (1578.4 - 5887.3) | 3967.7 ± 4100  (1341 - 5998.2) | 4064.5 ± 2026.6  (2329.2 - 5850.7) | 0.927; -96.83  [2031.15;-2224.8] |
| C3a, ng/ml, mean ± SD (IQR) | 3773.6 ± 2244.9  (1998.8 - 5366) | 3712.6 ± 2206.7  (2045.7 - 4305.1) | 3834.5 ± 2400.4  (2010.8 - 5605.3) | 0.871; -121.93  [1395.14;-1639] |
| free haemoglobin, mg/ml,  mean ± SD (IQR) | 43.4 ± 32.9 (18 - 60.3) | 42.5 ± 35.8 (18.3 - 61.6) | 44.3 ± 31.7 (20.6 - 58.1) | 0.876; -1.72 [20.54;-23.99] |
| free haemoglobin, mg/ml,  mean ± SD (IQR) | 6.2 ± 4.7 (3.1 - 8) | 6.1 ± 4.5 (2.9 - 8.3) | 6.2 ± 5.1 (3.4 - 7.7) | 0.985; -0.03 [3.14;-3.2] |
| free haemoglobin, mg/ml,  mean ± SD (IQR) | 6.4 ± 6.8 (2 - 7.3) | 5.1 ± 4.9 (2 - 5.9) | 7.6 ± 8.4 (2.2 - 8.4) | 0.267; -2.51 [1.99;-7.02] |
| Haptoglobine, mg/dl,  mean ± SD (IQR) | 645.7 ± 794  (299.3 - 670.8) | 574.2 ± 459.2  (314 - 687.5) | 717.3 ± 1053.1  (276 - 659.5) | 0.722^#^; -143.11  [391.45;-677.66] |
| Haptoglobine, mg/dl,  mean ± SD (IQR) | 43.9 ± 30.7 (19 - 70) | 45.3 ± 34.3 (19 - 64.5) | 42.6 ± 28.5 (19 - 68) | 0.791; 2.73 [23.49;-18.03] |
| Haptoglobine, mg/dl,  mean ± SD (IQR) | 89.7 ± 50.8 (39.3 - 135.5) | 87.1 ± 56 (35 - 135.5) | 92.4 ± 48.1 (51 - 135) | 0.753; -5.37 [28.96;-39.69] |
| Myoglobin, ng/ml, mean ± SD (IQR) | 45.9 ± 31.9 (19 - 60.8) | 46.8 ± 37.1 (19 - 59.5) | 45.1 ± 27.8 (22 - 57.5) | 0.737^#^; 1.73 [23.3;-19.84] |
| Myoglobin, ng/ml, mean ± SD (IQR) | 950.9 ± 1559.3 (185 - 694.5) | 1088.7 ± 1805.2 (215 - 689) | 813.1 ± 1354.4 (137 - 639) | 0.598; 275.63  [1325.66;-774.39] |
| Myoglobin, ng/ml, mean ± SD (IQR) | 897.1 ± 1779.5  (108.8 - 507.5) | 725.6 ± 1144.7  (125.5 - 537.5) | 1068.5 ± 2304.9  (94.5 - 356) | 0.566; -342.95  [854.43;-1540.33] |
| Fibrinogen, mg/dl, mean ± SD (IQR) | 268.6 ± 83.4 (220 - 300) | 258.3 ± 70.6 (225 - 290) | 278.4 ± 96.8 (225 - 305) | 0.476; -20.04 [36.33;-76.4] |
| Fibrinogen, mg/dl, mean ± SD (IQR) | 344.3 ± 104.3 (280 - 420) | 355 ± 111.9 (292.5 - 435) | 334.2 ± 101.5 (240 - 410) | 0.559; 20.79 [92.23;-50.66] |
| Fibrinogen, mg/dl, mean ± SD (IQR) | 460.5 ± 121.6 (382 - 550) | 487.9 ± 93.1 (417.5 - 557.5) | 434.5 ± 144 (375 - 540) | 0.188; 53.42 [133.93;-27.1] |
| Albumin, g/dl, mean ± SD (IQR) | 2.3 ± 0.5 (2 - 2.6) | 2.5 ± 0.5 (2.2 - 2.8) | 2.2 ± 0.4 (1.9 - 2.4) | 0.028; 0.35 [0.66;0.04] |
| Albumin, g/dl, mean ± SD (IQR) | 2.7 ± 0.5 (2.3 - 3) | 2.8 ± 0.4 (2.6 - 3.2) | 2.5 ± 0.5 (2.2 - 2.9) | 0.027; 0.34 [0.64;0.04] |
| Albumin, g/dl, mean ± SD (IQR) | 2.7 ± 0.4 (2.5 - 3) | 2.9 ± 0.3 (2.8 - 3.1) | 2.6 ± 0.5 (2.5 - 2.9) | 0.04; 0.29 [0.57;0.02] |
| Syndecan-1, ng/ml, mean ± SD (IQR) | 180.1 ± 103.6 (93.8 - 257.6) | 181 ± 97 (114.5 - 242.4) | 179.2 ± 115.2 (90.4 - 271) | 0.958; 1.83 [71.89;-68.23] |
| Syndecan-1, ng/ml, mean ± SD (IQR) | 201.3 ± 105.9 (116.4 - 268.2) | 201 ± 107.6 (113.1 - 270.9) | 201.6 ± 110 (124.5 - 260.2) | 0.987; -0.58 [71.01;-72.16] |
| Syndecan-1, ng/ml, mean ± SD (IQR) | 192.1 ± 113.6 (97.6 - 264.4) | 200.4 ± 109.4 (132.8 - 264.8) | 183.9 ± 123 (85.4 - 258.7) | 0.664; 16.52 [93.12;-60.07] |
| Hyaluronan, ng/ml, mean ± SD (IQR) | 198.3 ± 173.7  (109.7 - 190.9) | 165.5 ± 89  (110.6 - 170.1) | 231.2 ± 231.2  (110 - 208.8) | 0.919^#^; -65.68  [49.61;-180.97] |
| Hyaluronan, ng/ml, mean ± SD (IQR) | 543.3 ± 910.6  (145.6 - 410.5) | 615.7 ± 1220.7  (143.4 - 324.8) | 470.8 ± 499.3  (146.6 - 653) | 0.636; 144.87  [758.52;-468.78] |
| Hyaluronan, ng/ml, mean ± SD (IQR) | 957.2 ± 2308.5  (152.4 - 330.8) | 509.3 ± 802.4  (150.6 - 305.6) | 1405.2 ± 3191  (156 - 593.4) | 0.249; -895.93  [635.01;-2426.86] |
| Heparan sulfate, ng/ml,  mean ± SD (IQR) | 1611.3 ± 1115.5  (840.4 - 2113.2) | 2025.8 ± 1358.6  (929.2 - 2583.2) | 1196.9 ± 646.9  (760.5 - 1612.4) | 0.024^#^; 828.91 [1529.02;128.79] |
| Heparan sulfate, ng/ml,  mean ± SD (IQR) | 2058.5 ± 1572.7  (922.6 - 2999.2) | 2308.6 ± 1795.3  (1049.4 - 2978.5) | 1808.5 ± 1366.2  (671.4 - 2979.6) | 0.341; 500.03  [1549.69;-549.64] |
| Heparan sulfate, ng/ml,  mean ± SD (IQR) | 2538.5 ± 2509  (1069.2 - 2961.3) | 3188.8 ± 3096.1  (1338 - 3131.2) | 1888.2 ± 1676.5  (1012.2 - 2263.9) | 0.119; 1300.53  [2938.7;-337.64] |
|  |  |  |  |  |
| **Clinical outcome parameters** |  |  |  |  |
| MAP, mmHg, mean ± SD (IQR) | 77.8 ± 12.5 (70 - 89.3) | 81.3 ± 11.1 (73 - 88.5) | 74.2 ± 13.4 (64.5 - 87.5) | 0.084; 7.11 [15.22;-1.01] |
| MAP, mmHg, mean ± SD (IQR) | 75.4 ± 12.5 (67 - 83) | 76.3 ± 13.9 (69 - 86.5) | 74.5 ± 11.6 (67.8 - 78) | 0.669; 1.82 [10.37;-6.74] |
| MAP, mmHg, mean ± SD (IQR) | 75.9 ± 18.7 (63 - 87.5) | 73.3 ± 22.5 (60.8 - 80) | 78.4 ± 14.8 (67.5 - 88.5) | 0.432; -5.06 [7.85;-17.96] |
| CI l/min/m^2^(N=34 control =17, treatment =17), mean ± SD (IQR) | 2.4 ± 0.7 (1.9 - 2.9) | 2.3 ± 0.6 (2 - 2.8) | 2.4 ± 0.8 (1.8 - 3.1) | 0.608; -0.12 [0.36;-0.61] |
| CI, l/min/m2, mean ± SD (IQR)  (N=28, control=14, treatment=14) | 2.8 ± 0.5 (2.5 - 3.1) | 2.7 ± 0.5 (2.5 - 3) | 2.8 ± 0.7 (2.4 - 3.1) | 0.574; -0.12 [0.32;-0.56] |
| CI, l/min/m2, mean ± SD (IQR)  (N=15, control=6, treatment=9) | 2.9 ± 0.7 (2.4 - 3.3) | 2.4 ± 0.4 (2.2 - 2.7) | 3.2 ± 0.7 (2.5 - 3.4) | 0.012; -0.82 [-0.21;-1.44] |
| GEDI, ml/m^2^, mean ± SD (IQR) | 823.3 ± 266.1  (675.5 - 850.8) | 759.4 ± 104.6  (680 - 843) | 887.1 ± 361.7  (674 - 899) | 0.178; -127.71  [58.3;-313.72] |
| GEDI, ml/m2, mean ± SD (IQR) | 783.7 ± 181.6 (666 - 851) | 748.1 ± 163.8 (709.8 - 850.5) | 816.9 ± 202.4 (658 - 879) | 0.322; -68.79 [71.1;-208.68] |
| GEDI, ml/m2, mean ± SD (IQR) | 943 ± 196 (827.5 - 989) | 889.2 ± 103.8 (820.8 - 939.5) | 978.9 ± 248.4 (841 - 1012) | 0.355; -89.72 [111.22;-290.67] |
| EVLWI, ml/kg, mean ± SD (IQR) | 11.8 ± 7.4 (8.1 - 12.8) | 10.2 ± 4.4 (7.7 - 10.2) | 13.4 ± 9.6 (8.3 - 14.3) | 0.227; -3.18 [2.04;-8.4] |
| EVLWI, ml/kg, mean ± SD (IQR) | 10 ± 2.6 (8.5 - 10.7) | 9.4 ± 1.7 (8.6 - 10.6) | 10.6 ± 3.2 (8.7 - 12) | 0.186; -1.28 [0.64;-3.19] |
| EVLWI, ml/kg, mean ± SD (IQR) | 10.5 ± 2.6 (8.6 - 11.4) | 10.2 ± 2.6 (8.9 - 10.5) | 10.7 ± 2.8 (8.5 - 12.2) | 0.714; -0.53 [2.54;-3.6] |
| paO2/FiO2, mmHG, mean ± SD (IQR) | 364.3 ± 260.9 (216.3 - 395) | 374.8 ± 264.5 (229.5 - 407.5) | 353.8 ± 271.1 (217.5 - 379.5) | 0.81; 21.05 [197.28;-155.17] |
| paO2/FiO2, mmHg, mean ± SD (IQR) | 270.2 ± 96.3 (203 - 302.5) | 291.2 ± 108.3 (210.5 - 322.5) | 249.2 ± 83.4 (200.5 - 274) | 0.189; 42 [105.57;-21.57] |
| paO2/FiO2, mmHg, mean ± SD (IQR) | 243.8 ± 97.4 (178 - 267) | 239.1 ± 49.9 (204 - 267.5) | 248.8 ± 134.1 (165 - 254.3) | 0.775; -9.73 [58.5;-77.96] |
| Laktat, mmol/l, mean ± SD (IQR) | 1.9 ± 1.2 (1.1 - 2.3) | 1.7 ± 0.7 (1.2 - 2) | 2.1 ± 1.5 (1.1 - 2.6) | 0.747^#^; -0.35 [0.45;-1.14] |
| Laktat, mmol/l, mean ± SD (IQR) | 2.4 ± 2.3 (1 - 2.5) | 2.2 ± 2.2 (1 - 1.9) | 2.7 ± 2.5 (1.4 - 3.4) | 0.461; -0.56 [0.97;-2.1] |
| Laktat, mmol/l, mean ± SD (IQR) | 2.6 ± 4 (1 - 1.7) | 1.2 ± 0.4 (1 - 1.4) | 3.7 ± 5.1 (1 - 3.7) | 0.089; -2.43 [0.32;-5.18] |
| PCT, ng/ml, mean ± SD (IQR) | 0.2 ± 0.4 (0.1 - 0.1) | 0.2 ± 0.2 (0.1 - 0.1) | 0.3 ± 0.5 (0.1 - 0.2) | 0.696^#^; -0.08 [0.17;-0.33] |
| PCT, ng/ml, mean ± SD (IQR) | 1.7 ± 3.2 (0.3 - 1) | 2.3 ± 4.3 (0.3 - 1) | 1 ± 1.6 (0.3 - 1.2) | 0.229; 1.29 [3.42;-0.83] |
| PCT, ng/ml, mean ± SD (IQR) | 2.2 ± 4.5 (0.2 - 1.2) | 3.3 ± 6.2 (0.3 - 2) | 1 ± 1.4 (0.2 - 1.2) | 0.128; 2.31 [5.26;-0.64] |
| SOFA –Score, mean ± SD (IQR) | 7.8 ± 1.9 (7 - 9) | 7.5 ± 1.9 (7 - 8.5) | 8.2 ± 2 (7 - 10) | 0.287; -0.68 [0.6;-1.97] |
| SOFA –Score, mean ± SD (IQR) | 6.4 ± 3.1 (4 - 9) | 6 ± 3.1 (4.5 - 8) | 6.8 ± 3.3 (4 - 10) | 0.423; -0.84 [1.26;-2.95] |
| SOFA –Score, mean ± SD (IQR) | 6.7 ± 3.3 (4 - 9) | 6.6 ± 3.3 (4.5 - 8.5) | 6.8 ± 3.6 (4 - 10) | 0.851; -0.21 [2.04;-2.46] |
| NuDesc Score, mean ± SD (IQR) | 1.2 ± 1.8 (0 – 2.5) | 1.3 ± 1.7 (0 - 3) | 1.1 ± 1.9 (0 - 1) | 0.673; 0.28 [1.63;-1.07] |
| number of patients needing noradrenaline, n (%) | 37 (97.4) | 19 (50) | 18 (47.4) | 0.999 |
| noradrenaline, flow;µg/kg/min (at 0.1 mg/ml), mean ± SD (IQR) | 0.1 ± 0.1 (0.1 - 0.2) | 0.1 ± 0.1 (0 - 0.2) | 0.2 ± 0.1 (0.1 - 0.2) | 0.328; -0.04 [0.04;-0.11] |
| duration of noradrenaline need, h,  mean ± SD (IQR) | 9.7 ± 5 (5 - 13) | 10.3 ± 5.9 (5.5 - 15.5) | 8.9 ± 3.8 (5 - 12) | 0.411; 1.38 [4.74;-1.98] |
| duration of noradrenaline need, duration, h, mean ± SD (IQR), n=20 | 18.6 ± 7 (10 - 24) | 16.5 ± 7.3 (10 - 24) | 21.1 ± 6.7 (22 - 24) | 0.168; -4.51 [2.08;-11.1] |
| number of patients needing adrenaline, n (%) | 5 (13.2) | 4 (10.5) | 1 (2.6) | 0.15 |
| adrenaline, flow;µg/kg/min (at 0.1 mg/ml), mean ± SD (IQR) | 0.1 ± 0.1 (0.1 - 0.1) | 0.1 ± 0.1 (0.1 - 0.2) | 0.1 ± 0 (0.1 - 0.1) | - |
| duration of adrenaline need, h,  mean ± SD (IQR), n=5 | 12.2 ± 3.9 (9 - 16) | 12.3 ± 5 (8.5 - 16.3) | 12 ± 0 (12 - 12) | - |
| duration of adrenaline need, duration, h, mean ± SD (IQR), n=4 | 23.3 ± 1.3 (23.3 - 24) | 24 ± 0 (24 - 24) | 22.5 ± 2.1 (21.8 - 23.3) | 0.5; 1.5 [7.95;-4.95] |
| number of patients needing dobutamine, n (%) | 13 (34.2) | 4 (10.5) | 9 (23.7) | 0.087 |
| dobutamine, flow;µg/kg/min (at 5 mg/ml), mean ± SD (IQR) | 4 ± 1.3 (3.3 - 5.3) | 2.9 ± 1.4 (2.3 - 4) | 4.5 ± 1.1 (3.7 - 5.6) | 0.123; -1.53 [0.27;-3.32] |
| duration of dobutamine need, h,  mean ± SD (IQR), n=16 | 12.4 ± 4.9 (11.3 - 16) | 13 ± 4.6 (12 - 16) | 12.1 ± 5.5 (11 - 16) | 0.738; 0.91 [6.56;-4.75] |
| duration of dobutamine need, duration, h, mean ± SD (IQR), n=16 | 17.4 ± 9.4 (6.3 - 24) | 23.3 ± 1.6 (24 - 24) | 13.8 ± 10.9 (4 - 24) | 0.022; 9.53 [17.04;2.03] |
| time of mechanical ventilation, h,  mean ± SD (IQR) | 9.9 ± 3.2 (7.5 - 12) | 10.1 ± 2.9 (7.8 - 11.5) | 9.7 ± 3.6 (6.8 - 12) | 0.744; 0.36 [2.57;-1.85] |
| time of mechanical ventilation, h,  mean ± SD (IQR), n=3 | 1 ± 0 (1 - 1) | 1± 0 (1 - 1) | 1 ± 0 (1 - 1) | - |
| number of needed transfusions,  mean ± SD (IQR) | 0.7 ± 1.3 (0 - 1) | 0.5 ± 0.8 (0 - 1) | 0.8 ± 1.7 (0 - 1.5) | 0.476; -0.32 [0.57;-1.2] |
| number of needed transfusions,  mean ± SD (IQR) | 1.1 ± 2.2 (0 - 2) | 0.7 ± 0.9 (0 - 2) | 1.5 ± 3 (0 - 2) | 0.262; -0.84 [0.64;-2.33] |
| IABP, n (%) | 2 (5.3) | 2 (5.3) | 0 (0) | 0.146 |
| IABP, n (%) | 2 (5.3) | 2 (5.3) | 0 (0) | 0.146 |
| ECMO vv, n (%) | 0 (0) | 0 (0) | 0 (0) | - |
| ECMO vv, n (%) | 0 (0) | 0 (0) | 0 (0) | - |
| ECMO va, n (%) | 0 (0) | 0 (0) | 0 (0) | - |
| ECMO va, n (%) | 1 (2.6) | 0 (0) | 1 (2.6) | 0.311 |
| SIRS development, n (%) | 7 (18.9) | 4 (10.8) | 3 (8.1) | 0.734 |
| SIRS development, n (%) | 6 (15.8) | 2 (5.3) | 4 (10.5) | 0.374 |
| Sepsis development, n (%) | 0 (0) | 0 (0) | 0 (0) | - |
| Sepsis development, n (%) | 0 (0) | 0 (0) | 0 (0) | - |
| crystalloid solution, ml,  mean ± SD (IQR) | 5454.9 ± 1632.7  (4378 - 6118.5) | 5967.1 ± 1690.1  (4746 - 7084) | 4942.6 ± 1489.1  (4096 - 5609.5) | 0.046^#^; 1024.47  [2072.53;-23.58] |
| crystalloid solution, ml, mean ± SD (IQR) | 3891.8 ± 1746.9  (2966 - 4442) | 3518.5 ± 1632.1  (2967.5 - 4027.5) | 4245.4 ± 1867  (2968 - 5296) | 0.215; -726.87  [441.9;-1895.64] |
| colloid solution, ml, mean ± SD (IQR) | 65.8 ± 204.3 (0 - 0) | 26.3 ± 114.7 (0 - 0) | 105.3 ± 267.7 (0 - 0) | 0.249; -78.95 [56.54;-214.43] |
| colloid solution, ml, mean ± SD (IQR) | 0 ± 0 (0 - 0) | 0 ± 0 (0 - 0) | 0 ± 0 (0 - 0) | - |
| urine volume per 24h, ml,  mean ± SD (IQR) | 1514.4 ± 835.8  (926.3 - 2105) | 1724.7 ± 955.7  (1265 - 2235) | 1304.2 ± 684.1  (855 - 1865) | 0.129; 420.47  [967.3;-126.36] |
| urine volume per 24h, ml,  mean ± SD (IQR) | 2498.2 ± 1683.5  (1290 - 3190) | 2361.7 ± 1502.3  (1167.5 - 3155) | 2627.5 ± 1912.6  (1430 - 3215) | 0.64; -265.86  [878.8;-1410.52] |
| total fluid balance, ml,  mean ± SD (IQR) | 3932.7 ± 1733  (2798.5 - 5053.8) | 4288.2 ± 1844.6  (3473.5 - 5949) | 3577.2 ± 1634.4  (2584 - 4844.5) | 0.217; 711  [1857.68;-435.68] |
| total fluid balance in ml,  mean ± SD (IQR) | 1402.6 ± 2407.1  (10 - 2342) | 1214.4 ± 2406.5  (27.5 - 1878.3) | 1581 ± 2524.2  (-48 - 2612.5) | 0.654; -366.61  [1279;-2012.22] |
| renal replacement therapy needed,  n (%) | 3 (7.9) | 2 (5.3) | 1 (2.6) | 0.547 |
| renal replacement therapy needed,  n (%) | 5 (13.2) | 2 (5.3) | 3 (7.9) | 0.631 |

ICU admission

Day 1

Day2

Abbreviations: CI, cardiac index; ECMO, extracorporeal membrane oxygenation; EVLW, extravascular lung water; GEDI, global end-diastolic index; IABP, intra-aortic balloon pump; ICU, intensive care unit; IL, Interleukine; IQR, interquartil range; MD, mean difference; MAP, mean arterial pressure; NuDesc, Nursing Delirium Screening; PCT, procalcitonin; SD, standard deviation; SIRS, systemic inflammatory response syndrome; SOFA, Sepsis-related organ failure assessment score; TNF, tumor necrosis factor.

T-test, chi-square or fishers-exact test in case of n<5, ^#^ Mann Whitney-U in case of not normal distribution, ^a^ N=38, control n=18
