# Supplementary material for: REmoval of cytokines during CArdiac surgery (RECCAS): a randomised controlled trial
Source: Crit Care. 2024 Dec 12;28:406. doi: 10.1186/s13054-024-05175-9 (PMC11639119; doi:10.1186/s13054-024-05175-9)
Supplement: Supplementary file 6 — Supplementary Material 6. [file 13054_2024_5175_MOESM6_ESM.docx]

**Table follow-up**

|  | **Total** | **control** | **treatment** | **p-value; MD [CI]** |
| --- | --- | --- | --- | --- |
| **Number (n)** | **38** | **19** | **19** |  |
| number of secondary adverse events, n (%) | 4 (10.5) | 1 (2.6) | 3 (7.9) | 0.29 |
| length of ICU stay, h,  mean ± SD (IQR) | 2.8 ± 1.9 (1 - 4) | 2.8 ± 2.3 (1 - 4) | 2.7 ± 1.5 (1 - 4) | 0.87; 0.11 [1.4;-1.19] |
| number of ICU re-admissions, n (%) | 2 (5.3) | 1 (2.6) | 1 (2.6) | 1 |
| length of hospital stay, h,  mean ± SD (IQR) | 15 ± 10.3 (10 - 15.8) | 16.2 ± 12.7 (9.5 - 16.5) | 13.8 ± 7.8 (10 - 15.5) | 0.484; 2.42 [9.36;-4.51] |
| number of deceased, until discharge n (%) | 4 (10.5) | 1 (2.6) | 3 (7.9) | 0.29 |
| renal replacement therapy needed,  n (%) | 6 (15.8) | 3 (7.9) | 3 (7.9) | 1 |
| duration of renal replacement therapy, d, mean ± SD (IQR) | 3.8 ± 1.7 (2.3 - 5.5) | 5.3 ± 1.2 (5 - 6) | 2.3 ± 0.6 (2 - 2.5) | 0.029; 3 [5.07;0.93] |
| total time of mechanical ventilation, h, mean ± SD (IQR) | 16.6 ± 16.5 (7.1 - 15.3) | 15.4 ± 16.9 (7.8 - 12.3) | 17.8 ± 16.8 (5.5 - 27) | 0.914^#^; -2.44 [8.59;-13.47] |
| total duration of noradrenaline, h, mean ± SD (IQR), n=36 | 33.4 ± 34.1 (7.5 - 50.3) | 36.7 ± 38.7 (10 - 50.5) | 29.7 ± 30.1 (5 - 47) | 0.546^#^; 7 [28.91;-14.91] |
| total duration of epinephrine need, h, mean ± SD (IQR), n=6 | 63 ± 50.8 (23.8 - 76.3) | 65.8 ± 71.1 (13.8 - 99.5) | 57.5 ± 14.8 (52.3 - 62.8) | 0.624^#^; 8.25 [90.83;-74.33] |
| total duration of dobutamine need, h, mean ± SD (IQR), n=19 | 44.4 ± 38.6 (19.5 - 47.5) | 62 ± 55.4 (28.5 - 84) | 34.1 ± 24.2 (17.8 - 47.3) | 0.536^#^; 27.92 [62.81;-6.97] |
| total number of needed transfusions, mean ± SD (IQR), n=24 | 4 ± 4.8 (2 - 4) | 2.9 ± 1.9 (2 - 3) | 4.9 ± 6.4 (2 - 4) | 0.590^#^; -2.01 [2.8;-6.83] |
| total amount of needed fibrinogen, g, mean ± SD (IQR) | 1.4 ± 3.3 (0 - 0) | 1.1 ± 1.7 (0 - 2) | 1.8 ± 4.5 (0 - 0) | 0.447^#^; -0.74 [1.89;-3.36] |
| total volume of needed albumin, ml, mean ± SD (IQR) | 39.5 ± 142.4 (0 - 0) | 36.8 ± 160.6 (0 - 0) | 42.1 ± 130.5 (0 - 0) | 0.999^#^; -5.26  [85.26;-95.78] |
| total urine volume, ml,  mean ± SD (IQR) | 6200.8 ± 4926.6  (3732.5 - 7745.5) | 6041.2 ± 5731.9  (3075 - 7134.5) | 6360.4 ± 4281.5  (4065 - 8840) | 0.624^#^; -319.21  [2740.96;-3379.38] |
| total fluid balance, ml,  mean ± SD (IQR) | 6151.7 ± 6561.3  (2763.8 - 7543.3) | 6502.5 ± 6185.3  (3455 - 7510.5) | 5800.8 ± 7236.5  (1926 - 8764.5) | 0.624^#^; 701.68  [5274.24;-3870.87] |
